# Dissection of a grain yield QTL from wild emmer wheat reveals sub-intervals associated with culm length and kernel number

**DOI:** 10.3389/fgene.2022.955295

**Published:** 2022-10-19

**Authors:** Mathieu Deblieck, Gergely Szilagyi, Fatiukha Andrii, Yehoshua Saranga, Madita Lauterberg, Kerstin Neumann, Tamar Krugman, Dragan Perovic, Klaus Pillen, Frank Ordon

**Affiliations:** ^1^ Julius Kühn Institute (JKI), Federal Research Centre for Cultivated Plants, Institute for Resistance Research and Stress Tolerance, Quedlinburg, Germany; ^2^ The Hebrew University of Jerusalem, Robert H. Smith Faculty of Agriculture, Food and Environment, Rehovot, Israel; ^3^ University of Haifa, Institute of Evolution, Haifa, Israel; ^4^ Research Group Genebank Documentation, Leibniz Institute of Plant Genetics and Crop Plant Research (IPK) Gatersleben, Seeland, Germany; ^5^ Department of Plant Breeding, Martin-Luther-University Halle-Wittenberg, Institute of Agricultural and Nutritional Sciences, Halle, Germany

**Keywords:** wild emmer, grain yield, drought tolerance, culm length, kernel number, GBS, 15K-iSelect

## Abstract

Genetic diversity in wheat has been depleted due to domestication and modern breeding. Wild relatives are a valuable source for improving drought tolerance in domesticated wheat. A QTL region on chromosome 2BS of wild emmer wheat (*Triticum turgidum* ssp. *dicoccoides*)*,* conferring high grain yield under well-watered and water-limited conditions, was transferred to the elite durum wheat cultivar Uzan (*T. turgidum* ssp. *durum*) by a marker-assisted backcross breeding approach. The 2B introgression line turned out to be higher yielding but also exhibited negative traits that likely result from trans-, cis-, or linkage drag effects from the wild emmer parent. In this study, the respective 2BS QTL was subjected to fine-mapping, and a set of 17 homozygote recombinants were phenotyped at BC_4_F_5_ generation under water-limited and well-watered conditions at an experimental farm in Israel and at a high-throughput phenotyping platform (LemnaTec-129) in Germany. In general, both experimental setups allowed the identification of sub-QTL intervals related to culm length, kernel number, thousand kernel weight, and harvest index. Sub-QTLs for kernel number and harvest index were detected specifically under either drought stress or well-watered conditions, while QTLs for culm length and thousand-kernel weight were detected in both conditions. Although no direct QTL for grain yield was identified, plants with the sub-QTL for kernel number showed a higher grain yield than the recurrent durum cultivar Uzan under well-watered and mild drought stress conditions. We, therefore, suggest that this sub-QTL might be of interest for future breeding purposes.

## Introduction

The meteorological concept of drought can be defined as “an extended period of time characterized by a deficiency in a region’s water supply” (Below et al., 2007). Stress might be defined as an altered physiological condition that alters an equilibrium, leading to a strain, i.e., a biochemical and/or physical change, which can lead to injury, disease, or aberrant physiology of the plant (Gaspar et al., 2002). Plants can experience drought stress either when water supply becomes limited or when the transpiration rate becomes too high (Fahad et al., 2017). Drought stress effects on plants are, e.g., the reduction of water content, diminished leaf water potential and turgor loss, closure of stomata, decrease in cell enlargement/growth due to low turgor pressure, and arrest of photosynthesis, which can finally result in the disturbance of metabolism and death. Increased periods of drought were shown to have major negative effects on the yield (Lesk et al., 2016; Fahad et al., 2017). Potential yield losses differ according to the magnitude of stress and the developmental stage of the plant. Extreme yield losses were reported to be up to 92% (Farooq et al., 2014). Plant responses to drought stress can be divided into long- and short-term responses on the biochemical, molecular, and finally physiological levels. These responses lead to different strategies to deal with drought stress, i.e., escape, tolerance, avoidance, and recovery (Chen et al., 2015; Fang and Xiong, 2015).

Wheat (*Triticum* spp.) is among the top five grown crops in the world (Oyewole 2016; FAO 2019) and provides about 20% of the total calories consumed by humans (Poole et al., 2021). Future plant production faces the challenge of feeding the growing global population, which is predicted to reach about 9.8 billion people by 2050 (UN 2017). This challenge is increased by climate change, because per degree-Celsius increase in the global mean temperature wheat yield will be decreased by 6.0% (Fahad et al., 2017; Zhao et al., 2017). Developing high-yielding cultivars under drought with yield stability between environments is, thus, of prime importance (Langridge and Fleury, 2011).

The genetic diversity of tetraploid and hexaploid wheat has been depleted by the limited number of allopolyploidization events, domestication, and modern breeding (Dempewolf et al., 2017). Using crop wild relatives (CWR) such as wild emmer wheat in pre-breeding turned out to be an efficient tool to exploit their genetic diversity (Xie and Nevo, 2008; Huang et al., 2016; Dempewolf et al., 2017). A significant increase in the use of CWR has been noted since 1980 (Dempewolf et al., 2017). However, since abiotic stress tolerance is complex, the introgression of quantitative trait loci (QTL) from CWR aiming to confer tolerance to abiotic stress into elite cultivars is difficult (Xie and Nevo, 2008; Dempewolf et al., 2017). Traits that are advantageous for the CWR’s fitness can be detrimental for breeding purposes. An increased culm length, for instance, might allow the plant to compete for light (Lane et al., 2000) or to promote the distribution of the plants’ own seeds in the ecosystem—even if this leads to a trade-off in grain yield (GY). Avoiding trans-, cis-, or linkage drag effects from the CWR is, therefore, one of the most difficult issues in pre-breeding.

In 2017, the near-isogenic line NIL-U-2B-1 carrying a wild emmer QTL region on chromosome 2BS was shown to produce more GY than its recurrent elite durum parent, under water-limited and well-watered conditions (Merchuk-Ovnat et al., 2016; Merchuk-Ovnat et al., 2017). It also turned out that negative traits such as an increased culm length or delayed heading were introduced, possibly due to trans-, cis-, or linkage drag effects from the CWR (Merchuck-Ovnat et al., 2016). A recent analysis of NIL-U-2B-1 revealed that more than two-thirds of chromosome 2B and additional fragments of chromosomes 2A, 3A, and 5A were also introgressed from the donor wild emmer parent (Deblieck et al., 2020). Consequently, the original RIL mapping population, previously used for mapping the QTL on chromosome 2B, was re-genotyped with the 15k iSelect chip (TraitGenetics) to narrow down the previously identified QTL regions by a higher marker density of ∼4,000 SNPs (Fatiukha et al., 2021), which is much higher than the density used for the original mapping (Peleg et al., 2008; Peleg et al., 2009). The existence of the corresponding 20 cM large QTL-region for GY on chromosome 2BS was again confirmed under both water-limited and well-watered conditions (Fatiukha et al., 2021). Therefore, this study aimed at the fine mapping of a 15.67 cM region of this 20 cM large QTL, to identify the smallest sub-QTL region affecting grain yield, and to diminish the observed trans-, cis-, or linkage drag effects from the CWR. For this purpose, we used the iSelect marker Tdurum_contig27976_414 and wsnp_Ex_c6537_1133876, flanking the corresponding QTL-interval (Fatiukha et al., 2021) and 10 additional markers within the QTL, to establish a population of sub-NILs, carrying segmental chromosomal substitutions of the target region.

## Materials and methods

### Plant material

All plant materials were developed at the Hebrew University of Jerusalem (HUJI) in Rehovot (31°54′N, 34°47′E, 54 m above sea level). In past works, recombinant inbred lines (RILs), derived from a cross between durum wheat (cv. Langdon) and wild emmer wheat (acc# G18-16) were phenotyped under contrasting water availabilities and used for QTL mapping (Peleg et al., 2009; Fatiukha et al., 2021). Subsequently, the wild emmer QTL allele on chromosome 2BS conferring higher grain yield and harvest index (HI) was introgressed into the elite tetraploid durum wheat cultivar Uzan to develop the near-BC_3_F_3_ isogenic line (NIL) NIL-U-2B-1 as previously described (Merchuk-Ovnat et al., 2016; Deblieck et al., 2020). In this study, BC_3_F_3_ NIL-U-2B-1 (pollinator) was crossed with Uzan (Supplementary Figure S1) and BC_4_F_3_ plants were screened with the respective flanking molecular markers to identify heterozygous recombinants. The heterozygous recombinant BC_4_F_3_ plants were selfed and BC_4_F_4_ single seed descendants were again genotyped to identify homozygous recombinant plants for seed multiplication. Finally, BC_4_F_5_ seeds of 17 homozygous recombinant plants were used for phenotypic experiments in 2017, 2018, and 2019.

### Development of molecular markers and genotyping by sequencing (GBS)

The 15k iSelect data of G18-16, Langdon, Uzan, and NIL-U-2B-1 were previously published (Merchuk-Ovnat et al., 2016; Soleimani et al., 2020). Tdurum_contig27976_414 (32.88 cM) and wsnp_Ex_c6537_1133876/IAAV980 (48.55 cM) which flank the QTL-interval on chromosome 2B (Fatiukha et al., 2021) were converted into competitive allele-specific PCR (KASP) markers (Wilkinson et al., 2016). In addition to this, a total of 10 other PCR-based molecular markers, including KASP, cleaved amplified polymorphic sides (CAPS), and simple sequence repeats (SSRs) were developed along the QTL-interval at a distance of ∼1 cM to genotype and identify heterozygous recombinant BC_4_F_3_ sub-NILs. All molecular markers were tested and validated on the DNA of F_7_ lines of the original mapping population (Peleg et al., 2008; Peleg et al., 2009; Fatiukha et al., 2021). Furthermore, genotyping by sequencing (GBS) was applied to NIL-U-2B-1, the parental lines, the wild emmer wheat acc. G18-16, the durum elite parent Uzan, and homozygous sub-NILs with recombination close to the marker Gene-1741_103, which was previously described to be the nearest marker to the target QTL interval (Fatiukha et al., 2021). This additional step served to identify new recombination events within the recombinant sub-NILs to further improve fine-mapping efficiency.

GBS libraries were prepared as described previously (Elshire et al., 2011) and sequenced (150 bp paired-end, Illumina MiSeq). The sequencing produced millions of reads. These were de-multiplexed according to the barcodes and the adapters/barcodes using the CASAVA pipeline 1.8 (Illumina, Inc.). Trim Galore software from Babraham Bioinformatics (2012) was used for adapter and quality trimming of the amplified genomic sequences. After this first filtering, the trimmed sequences were aligned to the draft genome sequence of the wild emmer acc. Zavitan (v1) (Avni et al., 2017) using BWM-MEM (version: 0.7.7.-r1140) (Li, 2013), and variant-calling was performed with the samtool and bcftools (version: 0.1.19–96) (Li et al., 2009). High-quality bi-allelic SNPs were filtered, and the imputation of missing SNPs was conducted with Beagle (Browning and Browing, 2016). Aligned sequencing reads were used for SNP detection after a quality check (Q score >20). Multi-allelic SNPs, SNPs with minor allele frequency (MAF) < 5%, missing values ≥5%, or heterozygosity ≥90% were further excluded and a high-quality SNP genotyping dataset was compiled. SNPs which could clearly be assigned to a unique position on the physical genome of wild emmer were kept for analysis. GBS data were finally evaluated with the GenoTypeMapper (GTM) (Deblieck et al., 2020).

### Plant growth conditions and experimental design

A total of five plants per genotype were phenotyped under contrasting water-limited (WL) and well-watered (WW) conditions from 2017–2019.

In 2017 and 2018, plants were grown at the HUJI experimental farm at Rehovot. Seedlings were first placed in moist germination paper at 4C° in a dark vernalization room for 2 weeks and then transplanted at the beginning of December into an insect-proof screen house rain-protected by a polyethylene top. The soil was a brown-red sandy loam (Rhodoxeralf) composed of 76% sand, 16% clay, and 8% silt (Merchuk-Ovnat et al., 2016). The different water regimes were simulated in a factorial (genotype x irrigation regime) split plot block design. Each block consisted of two main plots (for the two irrigation regimes), split into subplots for genotypes. Each subplot consisted of a single row with five plants, 10 cm apart (50-cm long plots). In each bed, two 40 cm spaced rows were planted with 100 cm between each pair of rows (Supplementary Figure S2). Seasonal rainfall was simulated by applying water once or twice a week from planting in December to heading in April/May, leading to a total seasonal water application of 350 mm (for WL conditions) and 650 mm (for WW conditions) in 2017. However, drought stress appeared to be mild in 2017. To increase drought stress, 384 mm and 201 mm of water were applied to the segmental sub-NILs in 2018. Plants that grew poorly in a certain area of the screen house were excluded from the experiment, keeping three replicates per genotype.

In July 2019, one plant per genotype was grown per pot on the high throughput phenotyping (HTP) facility (LemnaTec-129 Scanalyzer 3D) (http://www.lemnatec.com) of the Leibniz Institute of Plant Genetics and Crop Plant Research (IPK) in Gatersleben in Germany (Supplementary Figure S3). To avoid the confounding effect of flowering time on drought evaluation, sub-NILs with a much later flowering time were not included in the HTP experiment. The controlled greenhouse is described by Neumann et al. (2015) along with the potting procedure. In short, pots (2 L) were filled in a standardized way by weighing in the same amount of soil as a standard substrate (“Klasmann Substrate no. 2” (http://www.klasmann-deilmann.com)) allowing a slow development of drought as described by Neumann et al. (2015). After sowing, 7 g of fertilizer was added to each pot (19% N, 9% P_2_O_5_, and 10% K_2_O) to supply the plants with nutrients throughout the life cycle. After 10 days, the plants were thinned out to one plant per pot. Directly after the daily image recording, each pot was weighed individually and watered to a previously defined target weight according to Dhanagond et al. (2019). Plants were grown on the platform from sowing until maturity and watered daily to the corresponding target weights.

The aim of the experiment was to mimic as good as possible the average temperatures and slowly progressing drought conditions of the screen house experiment conducted in Israel. Drought thresholds for severe drought stress were defined as 20% plant-available water (PAW) based on the results in barley (Dhanagond et al., 2019), while mild drought was defined as 30% PAW. No drought stress was applied until 30 days after sowing (30 DAS). The temperature during this pre-drought phase was set to 12°C at night and 16°C during the day. Supplementary greenhouse lights provided light for 15 h per day. To simulate drought stress, irrigation of plants intended for stress treatment was changed 31 DAS from 90% PAW to 30% PAW (mild drought threshold) and the temperature was raised to 20°C during the day and 16°C at night. At 62 DAS, the temperature was further increased to 24°C during the day and 20°C at night. From 64 DAS, watering was reduced to 20% PAW (severe drought level). This drought level and temperature regime lasted until maturity. At maturity, the plants were subjected to a detailed assessment of agronomic traits. Furthermore, the raw images were inspected, and the date of heading (BBCH 55) was determined for each plant.

### Phenotypic data, statistical analysis, and QTL-detection

Table 1 summarizes the type of traits that were recorded at maturity for each single plant (replicate) at the HUJI experimental farm and HTP platform (LemnaTec-129 Scanalyzer 3D) in Gatersleben. All statistical analyses were performed using R (version 3.4.1). The data of each trait, replication, year, and treatment were inspected separately. First, extreme outliers were filtered out, if they fell outside of an interval of plus or minus three times the standard deviation of the mean. Then, quantile–quantile (QQ) plots and density plots were used to evaluate whether the data were normally distributed. Data points which were not normally distributed at the QQ-plots residuals were removed manually. Subsequently, arithmetic means of the traits were calculated for each year/treatment and Shapiro–Wilk tests (Shapiro and Wilk, 1965) were carried out to confirm that data were normally distributed and suitable for further statistical analyses and tests. T-tests and ANOVA were applied to prove for each trait and/or year whether and to what extent they differ under both conditions. Descriptive statistics, i.e., density and correlation plots were calculated to analyze how the traits changed and correlate under the respective irrigation regimes. Finally, mean values of each trait, year, and treatment were used separately to calculate QTLs for each irrigation regime with the software MultiQTL2.6 (http://www.multiqtl.com).

**TABLE 1:** Phenotypic traits.

Trait^a^	Description
Calculated kernel number (CKN)	(GY/TKW)x1000
Culm length (CL)	Height from soil to the base of the spike in centimeter (cm)
Days planting to heading (DPH)	Days from planting to heading of 50% of the plants per plot in 2017
Grain yield (GY)	Grain yield in gram (g)
Harvest index (HI)	Ratio between grain yield and total dry weight
Thousand kernel weight (TKW)	TKW of all spikes (g)—including the main spike
Total biomass (TBM)	Total biomass per plant (g)
Main spike length (MSpL)	Main ear length without awns (cm)
Main spike thousand kernel weight (MSpTKW)	TKW of the main spikes (g)
Main spike spikelets (MSpSp)	Number of spikelets of the main ear
Main spike seeds per spikelet (MSpSpSp)	Number of seeds per spikelet of the main spike
Spikes per plant	Number of spikes per plant

a Please note that for each genotype, five plants were analyzed under drought stress and controlled conditions. Each of the trait was collected per replicate/plant. Subsequently, the means of the traits were calculated per genotype and conditions. For more details, please read the material and methods section of the article.

## Results

### Marker development

A total of 10 PCR-based molecular markers were developed at a distance of 1–2 centimorgan (cM) along the QTL-interval of 15.67 cM (Supplementary Tables S1-3). Within the interval, six additional polymorphic regions were identified by applying GBS to a subset of plants that showed recombinations close to the marker Gene-1741_103, thus, in between Kukri_c6227780 and Rac875_c2138_474 (Table 2). All markers share the same order in the genetic map and sequenced genome of the wild emmer acc. Zavitan (Table 2, Supplementary Table S1, Deblieck et al., 2020). For Tdurum_contig27976_414, no physical position could be annotated.

**TABLE 2:** Genotypic data of the recombinant inbred lines.

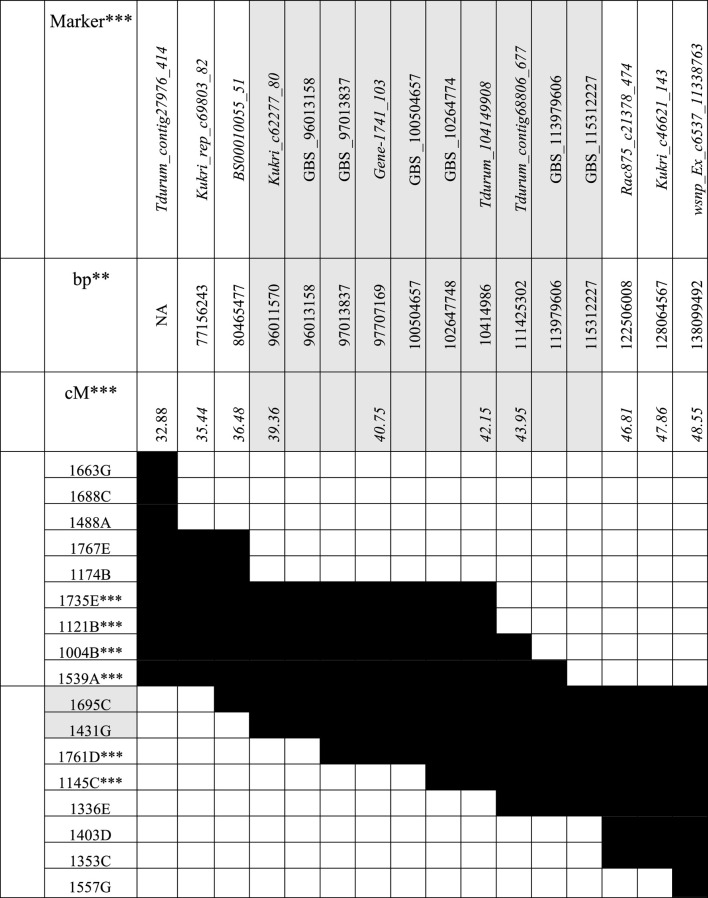

*15k iSelect markers that were used to develop PCR-based molecular markers are written in italics. Genotypic information that was obtained via GBS, is marked with “GBS” **. Physical positions were obtained from the wild emmer acc. Zavitan (v1) (Avni et al., 2017). Please note that no physical position was obtained for the marker Tdurum_contig27976_414. *** Genetic positions were obtained from the original mapping population (Fatiukha et al., 2021). **** Plants that showed recombination events between Kukri_c6227780 and Rac875_c2138_474 were subjected to GBS (please read material and methods section for more detailed information).

### Phenotypic data of homozygous recombinants sub-NILs

**FIGURE 1:**
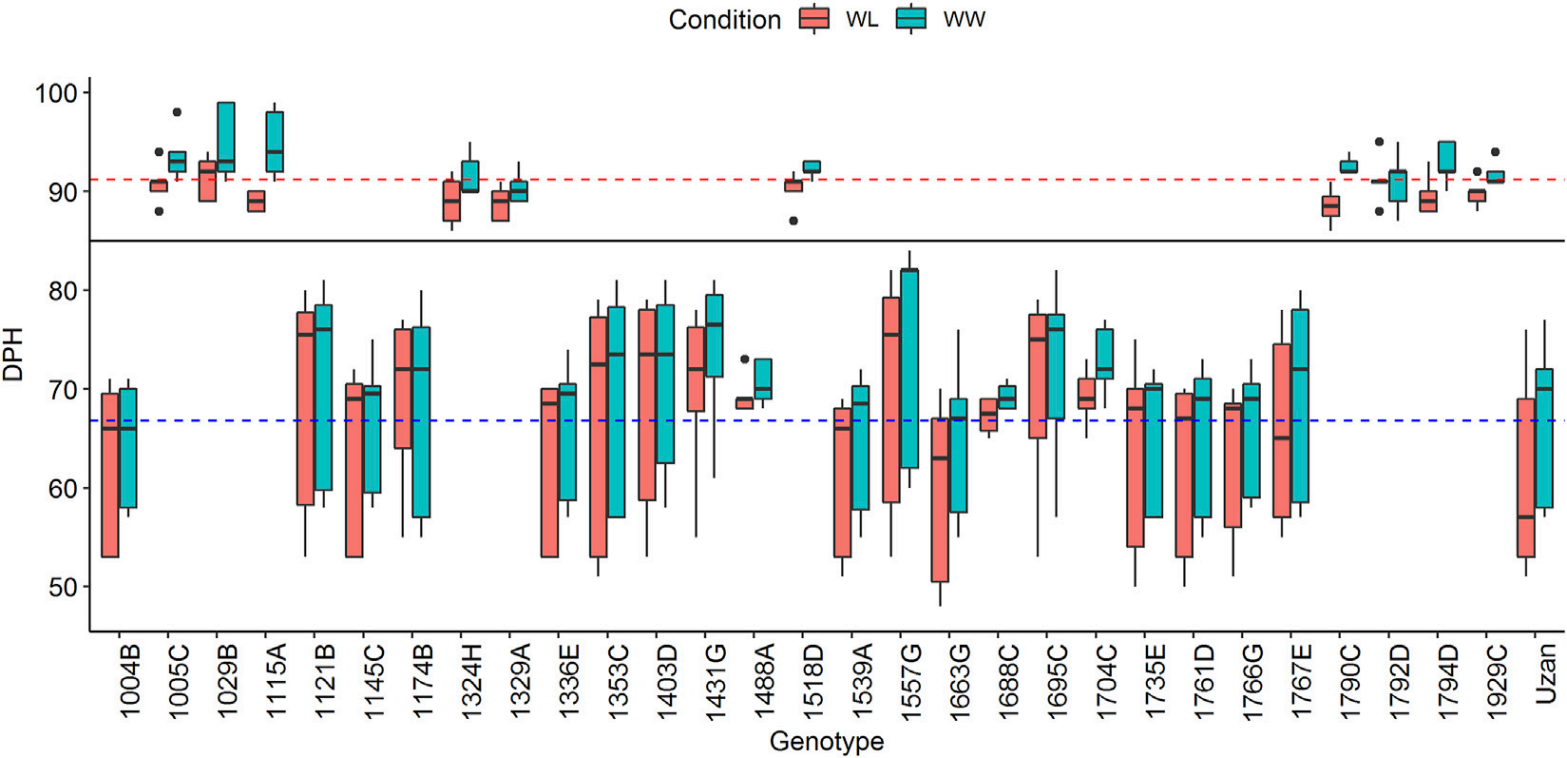
Days from planting to heading (DPH) of the complete set of 29 segmental sub-NILs in 2017. The black line indicates that plants cluster into two groups according to their DPH (for more details, see text). Blue and red dashed lines mark the mean DPH time of 66.79 and 91.22 days, respectively. Plants above the black line show delayed heading toward the elite parent Uzan and were, therefore, not considered for the phenotypic experiments in 2018 and 2019.

A total of 600 BC_4_F_3_ plants were derived from a cross of Uzan with NIL-U-2B-1 (Merchuk-Ovnat et al., 2016). Eighty-two heterozygous recombinant BC_4_F_3_ plants were identified after genotyping with the flanking Tdurum_contig27976_414 (32.88 cM) and wsnp_Ex_c6537_1133876/IAAV980 (48.55) markers (Supplementary Table S1). Subsequently, a sample of six to eight descendent plants of each heterozygote recombinant BC_4_F_3_ plant was screened and a total of 96 BC_4_F_4_ homozygote recombinants were obtained. After further genotyping the BC_4_F_4_ plants with the 10 markers (Supplementary Table S1, Supplementary Table S2, Supplementary Table S3), 29 of them with representative recombinations along the QTL interval were selected and used for seed multiplication. Finally, 10 plants per genotype, i.e., 290 BC_4_F_5_ plants, were subjected to phenotyping in 2017. Two very distinct groups of genotypes with different numbers of days from planting to heading (DPH) were observed (Figure 1). One group comprised 17 plants and required a mean time of 66.8 days for heading, whereas the other group comprised 12 plants and flowered 24.4 days later, thus, at 91.2 days. Grouping of these data was additionally confirmed with a Tukey’s test (Tukey, 1949) and independent segregation of DPH was confirmed with a chi-square test (Pearson, 1900). A comparison of GBS data of these plants revealed that sub-NILs with delayed DPH-values, i.e., 1029B, 1115A, 1329A, and 1929C, share a single 669 kbp fragment on chromosome 2A ranging from 39980886 to 40649713 BP, which is absent in early flowering sub-NILs, e.g., 1004B, 1539A, 1121B, 1663G, 1766G, 1761D, 1735F, 1336E, 1145C, and Uzan (Supplementary Table S4). As indicated previously, this region harbors the photoperiod sensitive gene *PpdA-1* from the wild emmer parent (Takenaka and Kawahara, 2012), ranging from 40487317–40489398 bp (Deblieck et al., 2020). Remarkably, this huge effect was not observed in the original mapping population (Fatiukha et al., 2021) although Langdon and Uzan share the same GBS-marker alleles along the PpdA-1 locus (Supplementary Table S4).

DPH correlates with grain yield (GY) and many important yield-related traits and might increase/decrease the respective QTL-LOD scores (Fatiukha et al., 2021). We, therefore, decided to exclude plants with increased DPH values from the phenotypic experiments in 2018 and 2019 (Figure 1). In addition to this, two sub-NILs did not deliver enough phenotypic data in 2018. For these genotypes, less than three replicates per genotype/treatment and year were available, which was considered unreliable and not representative, leading to a final set of 17 segmental sub-NILs. For each of these sub-NIL, 10 plants were subjected to phenotyping, i.e., phenotypic data were resolved for 170 plants in 2017, 2018, and 2019 (Table 2).

**FIGURE 2:**
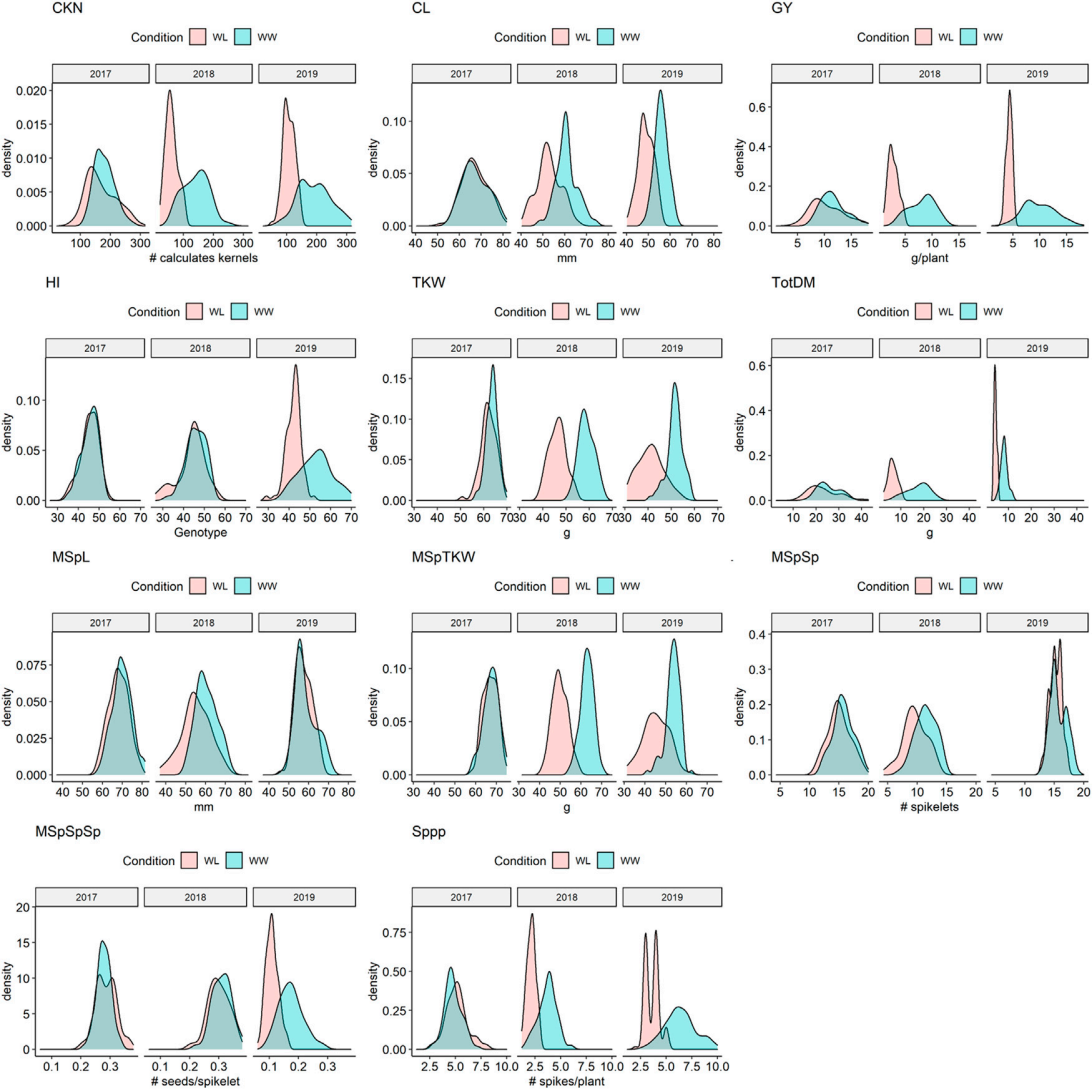
Density plots of selected traits under water-limited (WL) and well-watered conditions (WW) in 2017, 2018, and 2019. CKN = Calculated kernel number, CL = Culm Length, GY = Grain yield, HI = Harvest index, TKW = Thousand kernel weight, TotDM = Total Dry Matter, MSpL = Main Spike Length, MSpTKW = Main Spike thousand kernel weight, MSpSp = Main Spike Spikelets, MSpSpSp = Seeds per spikelet (of the main spike), Sppp = Spikelets per plant.

Almost all traits showed a normal distribution in both environments and all 3 years (Supplementary Table S5, Figure 2 and Supplementary Figure S4). However, in a few cases such as the main spike length (MSpL), the number of spikes per plant (Sppp), and grain yield per spike (GYpSp), phenotypic data were not normally distributed, and therefore, (log-) transformed (Hackett, 1997). These data are clearly highlighted, e.g., in Supplementary Table S5. ANOVA revealed that the means of all traits, except for the number of main spike spikelets (MSpSp), were different between the 3 years (Supplementary Table S6).

While in 2017 the drought stress effect was mild but significant for most of the traits except for culm length (CL), thousand kernel weight of the main spike (MSpTKW), and harvest index (HI), the effect of drought stress was very clear for all traits in 2018 and 2019 (Figure 2, Supplementary Figure S4, Supplementary Table S5).

**FIGURE 3:**
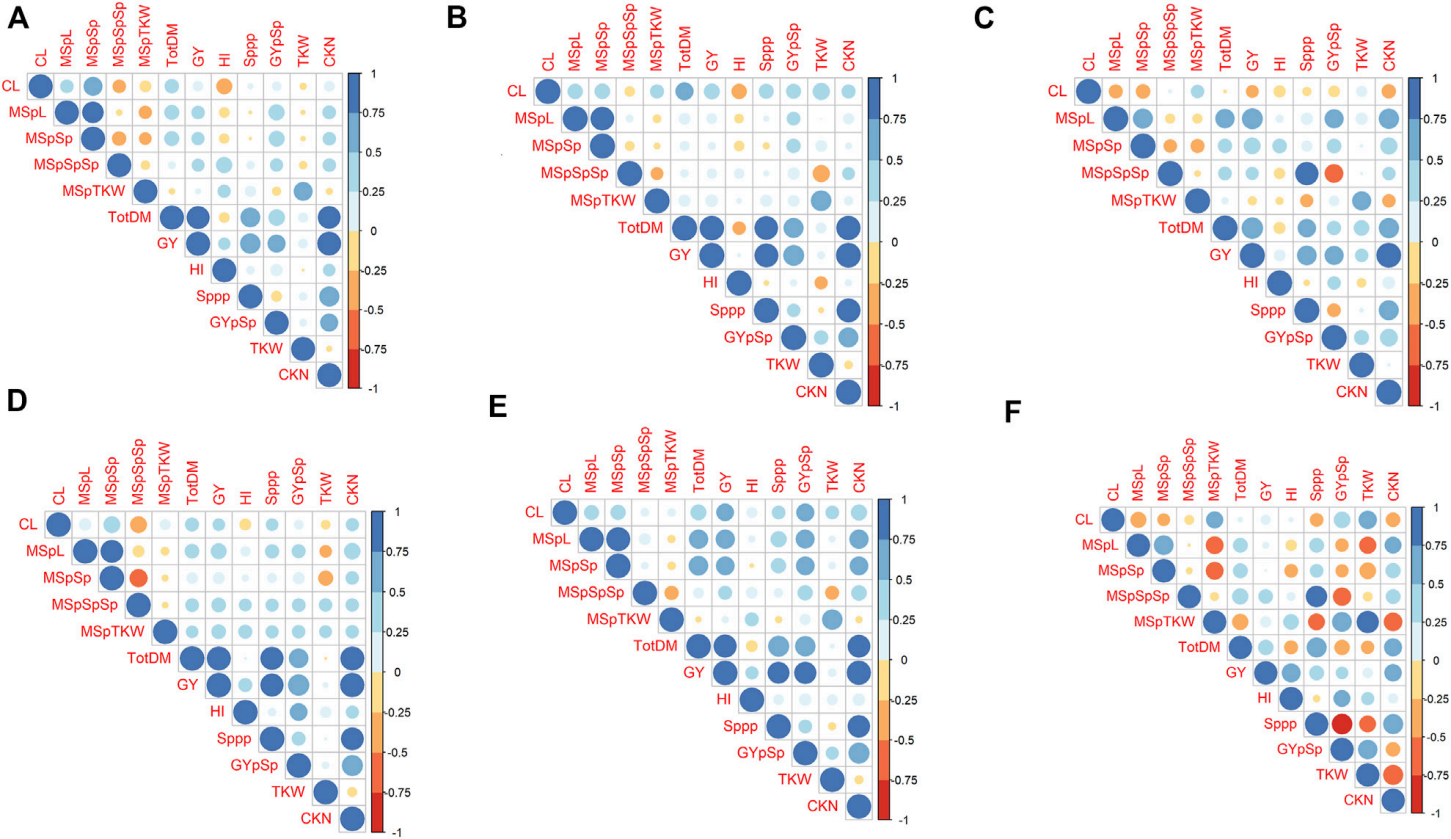
Correlation analysis of phenotypic data, obtained under water-limited (WL) and well-watered conditions (WW) in 2017, 2018 and 2019. Results for 2017, 2018 and 2019 were illustrated from the left to the right. The subplots **(A–C)** illustrate results that were obtained under WW conditions, **(D–F)** illustrate results for WL-conditions. CKN = Calculated kernel number, CL = Culm Length, GY = Grain yield, HI = Harvest index, TKW = Thousand kernel weight, TotDM = Total Dry Matter, MSpL = Main Spike Length, MSpTKW = Main Spike thousand kernel weight, MSpSp = Main Spike Spikelets, MSpSpSp = Seeds per spikelet (of the main spike), Sppp = Spikelets per plant.

In general, plants under drought stress have less GY, less MSpTKW, a reduced calculated kernel number (CKN), and shorter culms than under WW conditions (Figure 2 and Supplementary Figure S4, Supplementary Table S5, Supplementary Table S6). Traits such as CKN, MSpSp, MSpL, and the number of spikes per plant (Sppp) correlate most positively with GY under WW and WL conditions (Figure 3). In addition to this, HI correlates more negatively with CL and slightly more positively with CKN under WW conditions.

These drought stress effects were observed independently in 2018 and 2019. Genotypic and phenotypic data of all years and both water regimes are given in Supplementary Table S7.

### Sub-QTL-detection

Sub-QTLs under well-watered and water-limited conditions were calculated using data from 2017, 2018, and 2019, respectively. As mentioned previously, CL, MSpTKW, and HI did not show significant differences between drought and control conditions in 2017. Therefore, the data of these traits were not considered to calculate drought-specific QTLs. Table 3 shows some general information about the QTLs obtained, while Table 4 shows the logarithm of odds (LOD) values and dominance (d) effects of significant QTLs (*p* < 0.05) along the whole target interval. While QTLs for CL and TKW could be detected in both environments, QTLs for CKN or HI appear under drought stress or control conditions, respectively.

**TABLE 3:** Significant QTLs detected in 2017, 2018, and 2019.

	Year	2017					2018					2019						2017–2019				
Condition	Trait	LOD	P	Closest marker	PEV	Subst.	LOD	P	Closest marker	PEV	Subst.	LOD	P	Closest marker	PEV	Subst.	LOD		P	Closest marker	PEV	Subst.
						Effect					Effect					Effect						Effect
WL	CKN	2.39	0.01	*Kukri_rep_c69803_82*	0.50	24.60	1.96	0.06	*NOT SIGNIFICANT*	0.45	−13.67	1.81	0.08	*NOT SIGNIFICANT*	0.40	−17.85	2.59		0.01	*wsnp_Ex_c6537_1133876*	0.55	−13.00
	CL	*No significant difference between drought and control*					1.31	0.17	*NOT SIGNIFICANT*	0.37	−3.31	2.86	0.01	*wsnp_Ex_c6537_1133876*	0.54	3.88	2.88		0.01	*Kukri_rep_c69803_82*	0.65	−3.45
	HI						0.62	0.49	*NOT SIGNIFICANT*	0.17	−2.43	0.32	0.82	*NOT SIGNIFICANT*	0.10	1.17	1.33		0.19	*NOT SIGNIFICANT*		1.89
	MSpSp	0.46	0.66	*NOT SIGNIFICANT*	0.12	-1.01	0.70	0.46	*NOT SIGNIFICANT*	0.20	−0.88	2.55	0.02	*Kukri_c46621_14*	0.51	−1.03	2.24		0.03	*Tdurum_contig_68806_677*	0.60	−1.07
	MSpTKW	*No significant difference between drought and control*					2.95	0.01	*wsnp_Ex_c6537_1133876*	0.60	3.59	2.14	0.04	*Kukri_c46621_14*	0.45	6.47	1.14		0.20	*NOT SIGNIFICANT*	0.37	−3.38
	TKW	0.78	0.38	*NOT SIGNIFICANT*	0.20	2.41	3.86	0.00	*wsnp_Ex_c6537_1133876*	0.70	4.64	2.48	0.01	*wsnp_Ex_c6537_1133876*	0.49	6.04	2.67		0.01	*Kukri_rep_c69803_82*	0.61	−4.16
	CKNLOG	2.37	0.01	*Kukri_rep_c69803_82*	0.50	0.14																
WW	CL	3.01	0.01	*wsnp_Ex_c6537_1133876*	0.56	7.63	1.94	0.06	*NOT SIGNIFICANT*	0.46	4.14	2.34	0.00	*wsnp_Ex_c6537_1133876*	0.47	2.82	2.98		0.01	*Kukri_rep_c69803_82*	0.66	−4.20
	GYpSp	1.27	0.14	*NOT SIGNIFICANT*	0.29	−0.20	0.41	0.71	*NOT SIGNIFICANT*	0.12	−0.11	1.39	0.11	*NOT SIGNIFICAN*T	0.32	−0.22	1.64		0.06	*Kukri_rep_c69803_82*	0.40	0.16
	HI	3.67	0.00	*wsnp_Ex_c6537_1133876*	0.63	−5.36	1.50	0.11	*NOT SIGNIFICANT*	0.38	3.59	1.61	0.09	*NOT SIGNIFICANT*	0.36	7.91	2.69		0.01	*BS00010055_51*	0.56	6.00
	MSpL	1.00	0.25	*NOT SIGNIFICANT*	0.25	−2.92	0.58	0.53	*NOT SIGNIFICANT*	0.18	−2.77	2.41	0.02	*Tdurum_contig_68806_677*	0.55	−6.66	1.78		0.06	*Kukri_c6227780*	0.50	−3.73
	MSpSp	0.46	0.61	*NOT SIGNIFICANT*	0.12	−0.91	0.49	0.64	*NOT SIGNIFICANT*	0.16	−0.81	2.90	0.02	*Kukri_c46621_14*	0.55	−1.44	1.81		0.08	*NOT SIGNIFICANT*	0.55	−1.02
	MSpSpSp	1.65	0.08	*NOT SIGNIFICANT*	0.36	-0.02	2.75	0.02	*Kukri_rep_c69803_82*	0.64	0.05	0.61	0.50	*NOT SIGNIFICANT*	0.17	−0.02	2.33		0.03	*Gene-1741_103*	0.52	0.02
	TKW	1.15	0.17	*NOT SIGNIFICANT*	0.35	1.89	2.82	0.01	*Kukri_rep_c69803_82*	0.59	−4.08	1.62	0.04	24.90	0.38	−2.21	2.38		0.04	*Kukri_rep_c69803_82*	0.53	−1.96
	MSpLLOG											2.44	0.01	*Tdurum_contig_68806_677*	0.55	−0.11						

LOD, Logarithm of odds. The sign of the QTL, substitution effect d represents the effect of the G18-16 on the respective trait. It is the difference of the mean of homozygote Sub-NILs, with the G18-16 and Uzan-allele, respectively. CKN, calculated kernel number; CL, culm length; GY, grain yield; HI, harvest index; MSPL, main spike length; MSpSp, Main spike spikelets; MSpSpSp, Main Spike seeds per spikelet; MSpTKW, main spike thousand kernel weight; TKW, thousand kernel weight; TotDM, total dry matter; P, Probability (based on a permutation test with 1,000 repeats); PEV, Percentage of explained variance. Significant QTLs were highlighted in gray. In a few cases, the respective data of the year were not normal distributed and log transformed. These data were analyzed separately and written in italics. Log transformation was not successful in 2018 for the SPPP under WW and WL, conditions.

**TABLE 4:** QTL LOD-values along the target interval.

Condition	Year	iSelect	LOD-Values	Tdurum_contig27976_414	Kukri_rep_c69803_82	BS00010055_51	Kukri_c62277_80	GBS _96013158	GBS _97013837	Gene-1741_103	GBS _100504657	GBS _10264774	Tdurum_contig30989_79	Tdurum_contig68806_677	GBS_113979606	GBS_115312227	Rac875_c21378_474	Kukri_c46621_143	wsnp_Ex_c6537_1133876
		BP			77.16	80.47	96.01	96.01	97.01	97.70	100.50	102.65	10.41	111.43	113.98	115.31	122.51	128.06	138.10
		cM			35.44	36.48	39.36			40.75			42.15	43.95			46.81	47.86	48.55
WW	2017	CL	LOD		3.01	0.45	0.16	0.01	0.01	0.01	0.01	0.01	0.00	0.11	0.28	0.88	1.40	1.40	3.01
			eff.(d)		−7.63	−3.60	−2.11	0.40	0.55	0.55	0.55	0.55	−0.15	1.73	2.87	5.14	5.83	5.83	7.63
		HI	LOD		3.67	1.91	1.91	1.12	1.12	1.02	1.02	1.02	0.35	0.35	0.40	1.23	2.71	2.71	3.67
			eff.(d)		5.36	4.35	4.35	3.60	3.60	3.36	3.36	3.36	2.02	2.02	−2.26	−3.94	−4.94	−4.94	−5.36
	2018	CL	LOD		1.66	1.66	1.52	1.21	1.21	1.16	1.16	1.16	0.39	0.39	0.39	1.12	1.94	1.94	1.94
			eff.(d)		−3.90	−3.90	−3.79	−3.58	−3.58	−3.38	−3.38	−3.38	−2.04	−2.04	2.15	3.70	4.14	4.14	4.14
		MSpSpSp	LOD		2.75	1.73	1.54	1.17	1.23	1.23	1.23	1.23	0.65	0.65	0.05	0.09	0.52	0.52	1.65
			eff.(d)		0.05	0.04	0.04	0.03	0.03	0.03	0.03	0.03	0.02	0.02	−0.01	−0.01	−0.02	−0.02	−0.04
		TKW	LOD		2.82	2.82	1.63	0.66	0.66	0.40	0.40	0.40	0.21	0.21	0.10	0.25	1.34	1.34	1.34
			eff.(d)		−4.08	−4.08	−3.30	−2.39	−2.39	−1.83	−1.83	−1.83	−1.32	−1.32	0.96	1.60	3.11	3.11	3.10
	2019	CL	LOD		2.22	−1.43	−3.54	−0.42	−0.40	−0.42	−0.42	−0.44	0.01	0.97	1.19	1.63	1.63	1.56	2.34
			eff.(d)		−2.82	−1.70	−2.23	−0.90	−0.90	−0.84	−0.84	−0.84	0.17	2.00	2.25	2.69	2.69	2.45	2.82
		MSpL	LOD		0.60	0.00	0.84	0.84	1.30	1.30	1.30	1.98	1.98	2.41	1.99	1.47	1.79	1.79	1.79
			eff.(d)		3.50	0.17	−4.24	−4.24	−4.99	−4.99	−4.99	−5.82	-5.82	-6.66	−5.92	−5.67	−5.67	−5.67	−5.67
		MSpSp	LOD		2.64	0.91	0.91	0.03	0.03	0.00	0.00	0.07	0.07	0.61	0.64	1.34	2.90	2.90	2.90
			eff.(d)		1.48	0.92	0.92	0.18	0.18	−0.03	−0.03	−0.27	-0.27	−0.76	−0.80	−1.16	−1.44	−1.44	−1.44
		MSpLOG	LOD		0.64	0.00	0.83	0.83	1.30	1.30	1.30	1.99	1.99	2.44	2.05	1.53	1.81	1.81	1.81
			eff.(d)		0.06	0.00	−0.07	−0.07	−0.08	−0.08	−0.08	−0.10	−0.10	-0.11	−0.10	−0.10	−0.10	−0.10	-0.10
	2017 to 2019	CL	LOD		2.98	2.37	1.59	1.09	1.14	1.14	1.14	1.14	0.80	0.80	0.12	0.71	1.20	1.20	1.45
			eff.(d)		−4.20	−3.77	−3.20	−2.91	−2.86	−2.86	−2.86	−2.86	−2.41	−2.41	1.05	2.58	2.91	2.91	3.09
		HI	LOD		2.28	2.69	2.69	1.81	1.81	1.82	1.82	1.82	1.61	1.61	0.08	0.19	1.30	1.30	1.49
			eff.(d)		5.81	6.00	6.00	5.52	5.52	5.32	5.32	5.32	4.99	4.99	−1.31	−2.15	-4.68	−4.68	−4.84
		MSpSpSp	LOD		0.41	0.58	0.77	0.77	2.33	2.33	2.33	2.33	1.04	1.04	0.19	0.13	0.05	0.16	0.41
			eff.(d)		0.01	0.01	0.01	0.01	0.02	0.02	0.02	0.02	0.01	0.01	0.01	0.01	0.00	-0.01	−0.01
		TKW	LOD		2.38	2.38	0.89	0.82	0.82	0.34	0.34	0.34	0.13	0.13	0.13	0.54	1.49	1.49	1.56
			eff.(d)		−1.96	−1.96	−1.30	−1.33	−1.33	−0.86	−0.86	−0.86	−0.52	−0.52	0.55	1.18	1.65	1.65	1.65
WL	2017	CKN	LOD		2.39	2.39	1.97	0.15	0.15	0.00	0.00	0.03	0.03	0.11	0.16	0.36	0.47	0.47	0.88
			eff.(d)		24.60	24.60	22.23	7.04	7.04	0.99	0.99	2.97	2.97	−5.94	−7.24	−11.44	−11.91	−11.91	−15.73
		CKNLOG	LOD		2.37	2.37	1.99	0.20	0.20	0.01	0.01	0.05	0.05	0.11	0.16	0.39	0.47	0.47	0.90
			eff.(d)		0.14	0.14	0.13	0.05	0.05	0.01	0.01	0.02	0.02	−0.03	−0.04	−0.07	−0.07	−0.07	−0.09
	2018	MSpTKW	LOD		2.95	1.37	1.38	0.72	0.72	0.40	0.40	0.40	0.14	0.14	0.19	0.78	1.43	1.43	2.95
			eff.(d)		−3.59	−2.72	−2.84	−2.18	−2.18	−1.61	−1.61	−1.61	−0.95	−0.95	1.16	2.42	2.82	2.82	3.59
		TKW	LOD		3.86	2.69	2.29	1.04	1.04	0.28	0.28	0.28	0.17	0.17	0.21	0.92	2.60	2.60	3.86
			eff.(d)		−4.64	−4.25	−3.96	−3.08	−3.08	−1.63	−1.63	−1.63	−1.27	−1.27	1.48	3.11	4.20	4.20	4.64
	2019	CL	LOD		2.86	0.47	0.47	0.02	0.04	0.04	0.04	0.04	0.00	0.30	0.30	0.53	1.39	1.39	2.86
			eff.(d)		−3.88	−1.85	−1.85	−0.38	−0.56	−0.56	−0.56	−0.56	0.12	1.49	1.49	2.12	3.00	3.00	3.88
		MSpSp	LOD		1.67	0.28	0.24	0.24	0.24	0.22	0.22	0.46	0.46	1.10	1.10	1.45	2.55	2.55	2.55
			eff.(d)		0.87	0.41	−0.38	−0.38	−0.38	−0.35	−0.35	−0.49	−0.49	−0.74	−0.74	−0.90	−1.03	−1.03	−1.03
		MSpTKW	LOD		1.44	0.23	0.16	0.11	0.12	0.12	0.12	0.15	0.15	0.63	0.63	0.86	2.14	2.14	2.14
			eff.(d)		−5.47	−2.46	−1.99	1.72	1.74	1.74	1.74	1.89	1.89	3.87	3.87	4.81	6.47	6.47	6.47
		TKW	LOD		2.48	0.52	0.52	0.00	0.01	0.01	0.01	0.01	0.00	0.32	0.32	0.63	2.37	2.37	2.48
			eff.(d)		−6.04	−3.19	−3.19	−0.26	−0.36	−0.36	−0.36	−0.36	−0.01	2.51	2.51	3.74	6.03	6.03	6.04
	2017to 2019	CKN	LOD		2.59	2.45	2.45	0.34	0.34	0.11	0.11	0.11	0.02	0.33	0.33	0.81	1.58	1.58	2.59
			eff.(d)		13.31	12.77	12.77	5.81	5.81	3.20	3.20	3.20	1.46	−5.54	−5.54	−9.30	−11.08	−11.08	−13.00
		CL	LOD		2.88	1.94	1.85	0.68	0.68	0.55	0.55	0.55	0.31	0.31	0.23	0.73	1.32	1.32	1.47
			eff.(d)		−3.45	−2.91	−2.80	−1.96	−1.96	−1.70	−1.70	−1.70	−1.28	−1.28	1.17	2.16	2.51	2.51	2.57
		MSpSp	LOD		0.22	0.23	0.23	0.09	0.24	0.24	0.24	0.41	0.41	2.24	1.74	1.10	0.76	0.25	0.25
			eff.(d)		0.36	0.36	0.36	−0.24	−0.37	−0.37	−0.37	−0.48	−0.48	−1.07	−0.91	−0.78	−0.71	−0.38	−0.38
		TKW	LOD		2.67	2.45	2.28	0.77	0.77	0.41	0.41	0.41	0.18	0.31	0.31	0.48	1.55	1.55	1.55
			eff.(d)		−4.16	−3.93	−3.77	−2.57	−2.57	−1.87	−1.87	−1.87	−1.25	1.64	1.64	2.21	3.32	3.32	3.32

a LOD, logarithm of odds; d, sign of the QTL, substitution effect d represents the effect of G18-16 on the respective trait. It is the difference of the mean of homozygote sub-NILs, with the G18-16 and Uzan allele, respectively, CKN, calculated kernel number; GY, grain yield; HI, harvest index; MSPL, main spike length; TKW, thousand kernel weight; P, Probability (based on a permutation test with 1,000 repeats); PEV, Percentage of explained variance. No physical position was obtained for the marker Tdurum_contig27976_414. *** Genetic positions were obtained from the original mapping population (Fatiukha et al., 2021). Significant QTLs, with positive or negative effects on the trait and a LOD >2.0 are colored in yellow and blue.

**FIGURE 4:**
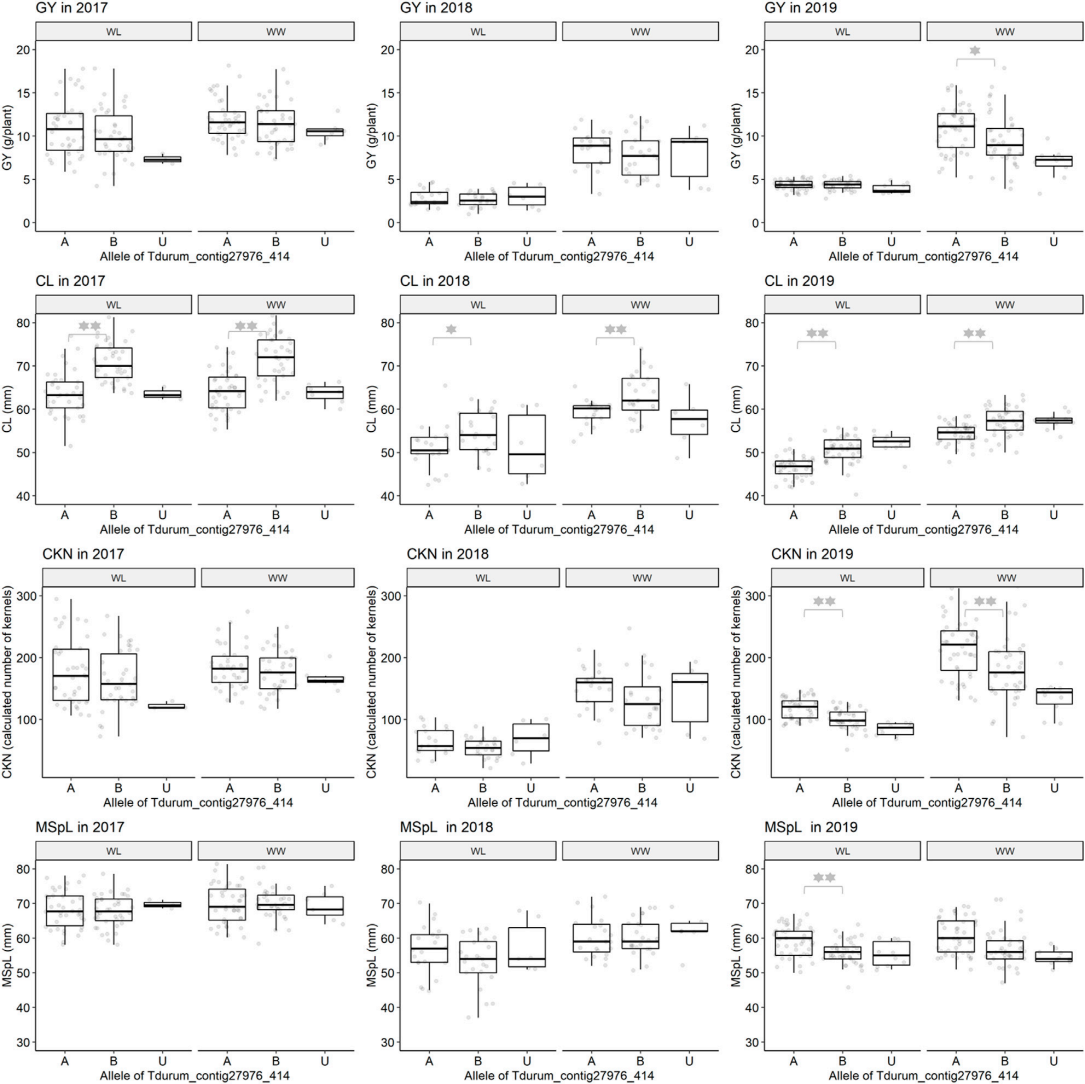
Grain yield (GY), culm length (CL), calculated kernel number (CKN), and main spike length (MSpL) of segmental sub-NILs with G18-16 or Uzan alleles of the iSelect marker Tdurum_contig27976_414. A and B Alleles were derived from G18-16 or Uzan, respectively. Values obtained for the elite parent Uzan (U) were illustrated separately. Significant differences between groups of genotypes with the different alleles of Tdurum_contig27976_414 were calculated with a two-sided *t*-test. *p*-values ≤ 0.05 or 0.01 were marked with one or two stars, respectively.

The QTLs for HI, CKN, and CL co-localize at the very distal parts of the QTL interval, leading to a biased set of segmental sub-NILs (i.e., northern or southern recombinants) which lack or harbor the respective part of the QTL (Table 2, Table 4 and Figure 4). Sub-NILs from the upper part of the wild emmer QTL tend to have shorter culms, an increased number of kernels, and slightly higher GY than sub-Nils than the lower part of the QTL (Table2; Figure 4).

**FIGURE 5:**
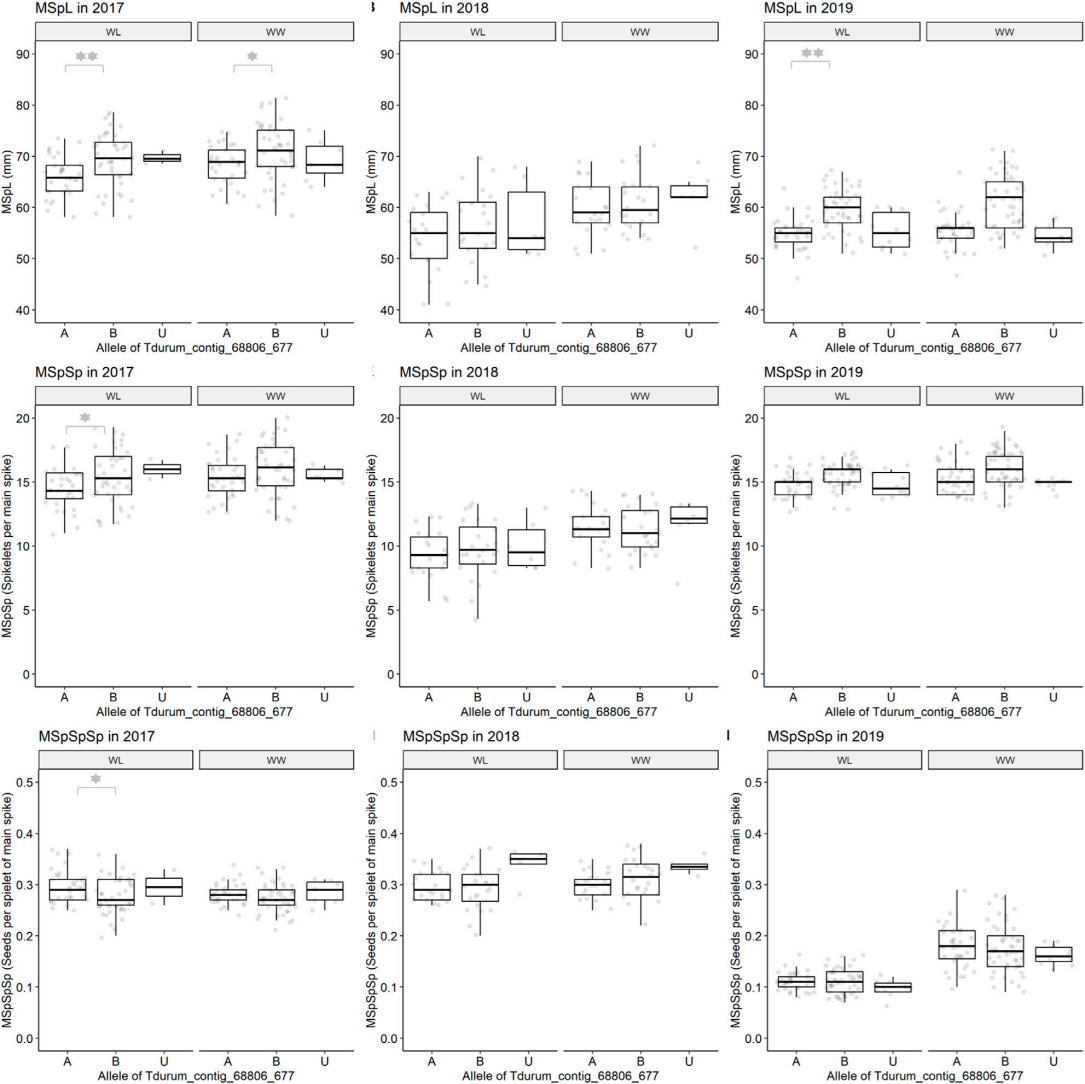
Main spike length (MSpL), main spike spikelets (MSpSp), and main spike seeds per spikelet (MSpSpSp) of segmental sub-NILs, carrying different alleles of the iSelect marker Tdurum_contig_68806_677. A and B Alleles were derived from G18-16 or Uzan, respectively. Values obtained for the elite parent Uzan (Uz) were illustrated separately. Significant differences between groups of genotypes with the different alleles of Tdurum_contig68806_677 were calculated with a two-sided *t*-test. *p*-values ≤ 0.05 or 0.01 were marked with one or two stars, respectively.

**FIGURE 6:**
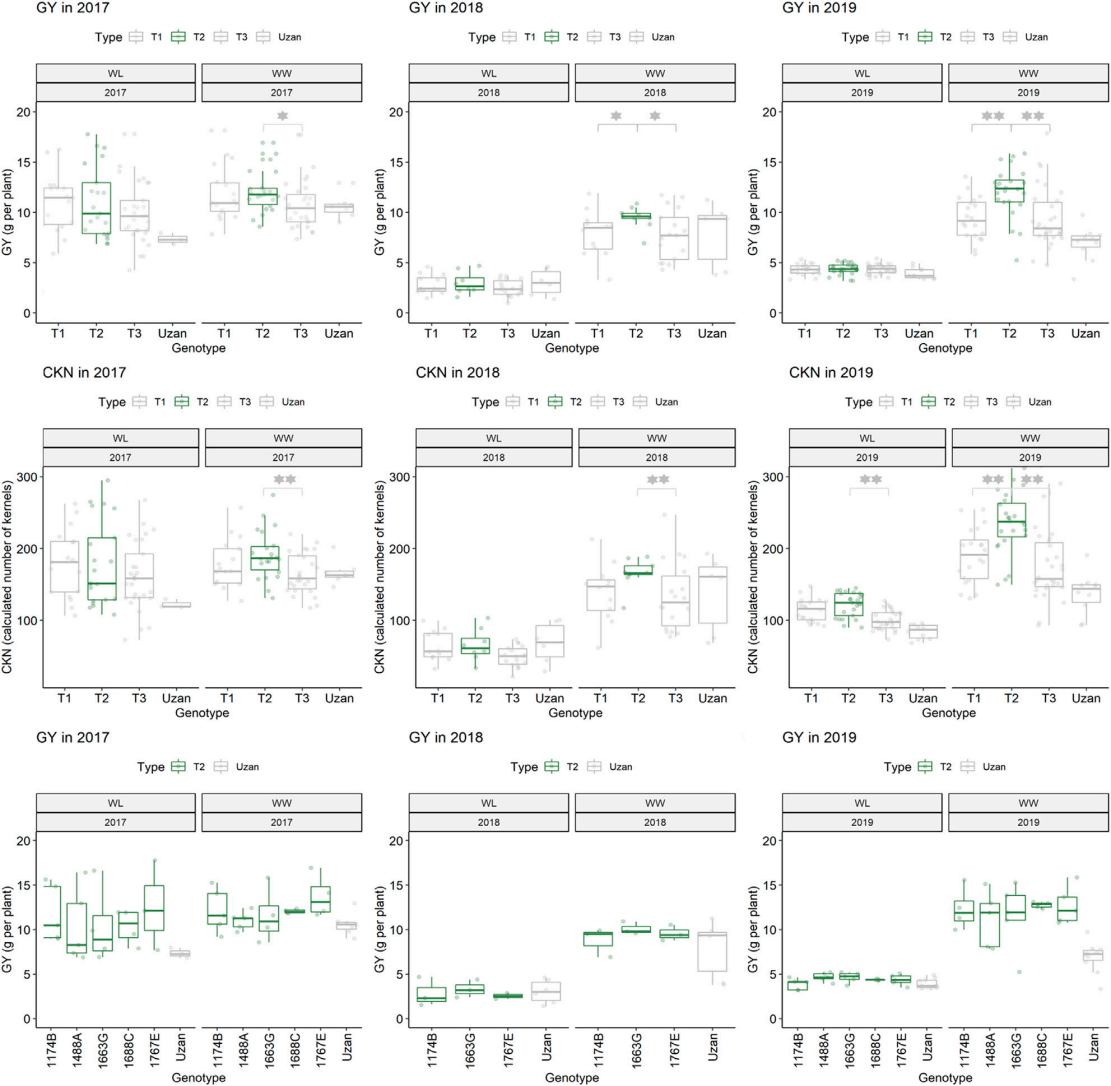
Grain yield (GY) and calculated kernel number (CKN) of segmental sub-NILs, carrying different alleles of the iSelect marker Tdurum_contig27976_414 and Gene-1741_103. T1: sub-NILs with the wild emmer allele from Tdurum_contig27976_414 and Gene-1741_103. T2 (green): sub-NILs with the wild emmer allele of Tdurum_contig27976_414 and elite parent allele of Gene-1741_103. T3: sub-NILs with the elite parent allele of Tdurum_contig27976_414 and Gene-1741_103. Uzan is the name of the respective elite parent. Figure C shows the GY values of each of the sub-NIL that belongs to T2. Significsant differences between the T1, T2, and T3 groups of genotypes were calculated with a two-sided *t*-test. *p*-values ≤ 0.05 or 0.01 were marked with one or two stars, respectively.

Furthermore, different QTLs related to traits of the main spike, e.g., for MSpSP, MSpTKW, and MSpL, were detected under WL and WW conditions between the markers Gene_1741_1 and Tdurum_contig_68806_677 (Table 4). Sub-NILs with the wild emmer allele of the marker Tdurum_contig_68806_677 appear to have a smaller main ear with fewer main ear spikelets (MSpSp) than the elite parent Uzan (Figure 5). Differentiating again between those sub-NILs which carry the upper part of the QTL interval and the G18-16 allele of the marker Tdurum_contig27976_414, but not the G18-16 allele of the flanking marker Gene-1741_103, revealed that these lines (i.e., 1663G, 1688C, 1488A, 1767E, and 1174B) again have a significantly higher HI, GY, and more CKN under WW conditions in all 3 years and under mild drought stress conditions in 2017 (Figure 6 and Table 4, Table 2). The average GY of these lines under WW conditions was 12.05 g (2017), 9.48 g (2018), and 12.07 g (2019), whereas Uzan yielded 10.60 g, 7.92 g, or 6.92 g (Table 5). The average GY of these lines under WW conditions in 2017, 2018, and 2019 was thus 12, 16, and 42% higher than of Uzan, respectively (Table 5).

**TABLE 5:** Mean values of segmental sub-NILs, carrying different alleles of the iSelect marker Tdurum_contig27976_414 and Gene-1741_103.

	Condition**	Year	Genotype^a^	Traits **											
				MSpSp	CL	MSpL	MSpSpSp	MSpTKW	TotDM	GY	HI	Sppp	GYpSp	TKW	CKN
Mean values	WL	2017	T1	14.40	63.71	66.68	0.32	67.96	22.83	10.96	48.72	5.28	2.03	62.38	178.67
			T2	14.81	63.49	69.38	0.29	66.36	23.51	11.00	46.53	5.06	2.06	61.85	178.45
			T3	15.22	70.43	68.18	0.27	66.01	23.67	9.89	42.27	5.23	1.87	61.92	160.88
			Uzan	16.00	63.60	69.73	0.30	65.51	16.75	7.35	44.41	4.63	1.59	59.96	122.46
		2018	T1	9.47	50.54	55.08	0.32	48.28	6.72	2.83	41.99	2.18	1.30	43.82	64.72
			T2	10.44	51.68	58.67	0.31	48.33	6.56	2.96	45.66	2.23	1.34	44.72	65.41
			T3	8.88	53.88	52.67	0.28	52.31	5.61	2.46	44.16	1.97	1.23	49.31	49.52
			Uzan	10.05	51.37	57.33	0.34	47.97	6.63	3.03	47.02	2.17	1.35	44.37	68.48
		2019	T1	15.30	47.99	55.60	0.10	43.90	4.17	4.32	41.55	3.55	1.24	38.17	113.90
			T2	15.92	45.55	62.28	0.11	40.48	4.32	4.38	42.18	3.92	1.14	36.73	121.92
			T3	14.90	51.27	56.45	0.11	46.94	4.24	4.33	42.09	3.66	1.21	43.42	100.27
			Uzan	14.80	52.10	55.40	0.10	51.40	3.63	3.94	41.82	2.90	1.37	46.85	84.15
	WW	2017	T1	15.12	64.36	67.53	0.29	68.55	24.71	11.83	49.09	4.55	2.53	63.57	177.60
			T2	15.53	64.06	71.46	0.28	66.05	25.78	12.05	46.79	4.77	2.49	63.22	190.80
			T3	15.95	70.39	70.33	0.26	67.05	25.15	10.69	41.52	4.71	2.24	63.41	164.74
			Uzan	15.60	63.67	69.24	0.29	69.45	21.31	10.60	49.84	4.75	2.26	63.44	167.01
		2018	T1	10.83	58.30	59.08	0.34	62.36	16.52	7.87	47.93	3.54	2.20	56.54	138.91
			T2	11.33	60.67	61.78	0.33	62.38	20.13	9.48	47.00	4.07	2.32	57.36	165.42
			T3	11.71	63.36	61.17	0.29	63.85	17.14	7.71	43.44	3.89	2.12	60.59	133.52
			Uzan	11.60	57.25	62.00	0.34	60.92	16.48	7.92	47.22	3.35	2.32	56.33	139.43
		2019	T1	16.20	54.83	56.65	0.16	52.37	7.45	9.38	58.98	6.25	1.51	49.36	189.82
			T2	16.58	54.03	63.58	0.17	53.24	8.77	12.07	55.45	6.79	1.76	51.72	235.38
			T3	15.03	57.69	57.67	0.18	54.48	8.31	9.37	49.60	6.40	1.50	52.79	177.00
			Uzan	14.80	57.28	54.60	0.16	54.70	6.86	6.92	45.51	5.30	1.41	51.70	137.42
*p*-values of a two sided t-test ***	WL	2017	T1 vsT2	0.54	0.90	0.12	0.02	0.16	0.77	0.97	0.06	0.57	0.74	0.71	0.99
			T2 vsT3	0.50	0.00	0.43	0.02	0.72	0.94	0.25	0.00	0.55	0.05	0.92	0.26
		2018	T1 vsT2	0.33	0.66	0.30	0.43	0.97	0.88	0.79	0.33	0.81	0.74	0.5	0.95
			T1 vsT3	0.13	0.39	0.09	0.03	0.01	0.36	0.27	0.63	0.12	0.37	0.00	0.09
		2019	T1 vsT2	0.02	0.00	0.00	0.44	0.06	0.44	0.7	0.74	0.05	0.09	0.25	0.11
			T2 vsT3	0.00	0.00	0.00	0.43	0.00	0.65	0.74	0.93	0.14	0.16	0.00	0.00
	WW	2017	T1 vs. T2	0.50	0.84	0.02	0.13	0.05	0.51	0.78	0.00	0.36	0.70	0.66	0.23
			T2 vsT3	0.46	0.00	0.45	0.01	0.35	0.62	0.04	0.00	0.78	0.00	0.78	0.01
		2018	T1 vsT2	0.55	0.05	0.30	0.41	0.99	0.03	0.05	0.6	0.07	0.22	0.38	0.07
			T2 vsT3	0.65	0.08	0.80	0.00	0.16	0.05	0.02	0.05	0.51	0.08	0.00	0.02
		2019	T1 vsT2	0.38	0.25	0.00	0.70	0.51	0.00	0.00	0.06	0.18	0.00	0.04	0.00
						T2 vsT3	0.00	0.00	0.00	0.26	0.17	0.24	0.00	0.00	0.36	0.01	0.19	0.00

a T1: sub-NILS, with the wild emmer allele from Tdurum_contig27976_414 and Gene-1741_103. T2: sub-NILS, with the wild emmer allele of Tdurum_contig27976_414 and elite parent allele of Gene-1741_103. T3: sub-NILS, with the elite parent allele of Tdurum_contig27976_414 and Gene-1741_103. Uzan: the name of the respective elite parent. ** GY, grain yield; CL , culm length, Sppp = Spikelets per plant, MSpL = main spike length, MSpSp = Main Spike Spikelets, MSpSpSp = Seeds per spikelet of the main spike, MSpTKW, main spike thousand kernel weight; CKN, calculated kernel number; CL, culm length; HI , harvest index; WW, Well-watered treatment; WL , Water-limited treatment *** *p*-values below 0.05 are highlighted in gray.

## Discussion and conclusion

In general, the following observations should be considered when comparing the results of this article to the previous studies by Peleg et al. (2009), Merchuk-Ovnat et al. (2016) and Fatiukha et al. (2021). First, it should be noted that the genetic background of the NIL-U2B-1 comes from cv. Uzan and not from cv. Langdon, the original parent of the mapping population (Peleg et al., 2009; Fatiukha et al., 2021). Although the effect of this QTL in NIL-U-2B-1 was confirmed previously (Merchuk-Ovnat et al., 2016), the different genomic backgrounds of Uzan against which the effect of the QTL region was determined in this study might have an impact on the detected QTLs. Second, the drought stress conditions that were used in 2018 and 2019 were probably more severe than in previous studies. Peleg et al. (2009) used 350 mm and Merchuk-Ovnat et al. (2016) used 360–290 mm of seasonal water application to mimic drought stress. In this study, 350 mm of seasonal water application was used only once in 2017 resulting in mild drought stress symptoms. About 201 mm of seasonal water application was used in 2018, and in 2019, water application was reduced from 64 DAS to 20% PAW to simulate severe drought stress conditions (Dhanagond et al., 2019). Average GY was significantly reduced under drought stress to 10.39 g, 2.75 g, and 4.33 g per plant in 2017, 2018, and 2019, respectively. Lower GY in 2018 and 2019 is very likely a consequence of increased drought stress. Furthermore, ANOVA analysis revealed that the means of all traits were different between all 3 years (Supplementary Table S6). This is probably due to different reasons. On the one hand, the temperatures in the screen houses in 2018 were very high, i.e., above 45°C (Supplementary Table S8). These high temperatures suppressed tillering and induced early heading so that the plants were shorter, and yield was lower than in 2017. On the other hand, the experimental setup in 2019 differed significantly from 2017 to 2018. Plants in the pot experiments at the HTP in 2019 appeared to be smaller, to have reduced biomass, and less TKW (Supplementary Table S6, Supplementary Figure S4). This may be due to environmental factors, e.g., the size of the pots and light intensity. However, as mentioned in the results section, the drought stress effect in 2017 was mild but significant for most of the traits, except for CL, MSpTKW, and HI, and very clear for all traits in 2018 and 2019 (Figure 2, Supplementary Figure S4, Supplementary Table S5).

In contrast to the previous studies by Peleg et al. (2009) and Fatiukha et al. (2020), no QTL for GY could be confirmed, but different segments of the introgressed wild emmer wheat region on chromosome 2B of NIL-U-2B-1 showed impacts on CL, CKN, and GY. The sub-NILs with the upper part of the emmer wheat QTL-interval had a shorter CL, more CKN, and a slightly higher GY, while sub-NILs with an introgression at the lower part of the QTL had longer stems, less CKN, and reduced GY (Figure 4; Table 4, Table 5). Furthermore, the central part of the QTL interval has a negative impact on MSPL and the number of MSPSP. Removing sub-NILs with this MSPL-QTL from the set of sub-NILs that harbor the upper part of the QTL interval leads to five sub-NILs (i.e., sub-NIL 1663G, 1688C, 1488A, 1767E, and 1174B) with significantly (*p* < 0.001) higher HI, CKN, and GY than Uzan (Table2, Table 5, Figure 6). The increased number of kernels seems to have a decisive effect on the GY of these five lines. This effect was significant under mild drought stress in 2017 and controlled conditions in 2017 and 2019, but not under more severe drought stress conditions in 2018 and 2019. It can, therefore, be stated that the upper part of the introgressed fragment on chromosome 2B in the sub-NILs 1663G, 1688C, 1488A, 1767E, and 1174B might be of value for future breeding programs. The observed increased GY under mild drought stress conditions in 2017 is consistent with the results of previous studies (Peleg et al., 2009; Merchuk-Ovnat et al., 2016; Fatiukha et al., 2021). It might, therefore, be hypothesized that this region has a yield stabilizing effect under mild drought stress conditions. However, further trials with these sub-NILs would be useful to characterize up to which degree of drought stress the effect of this introgression remains advantageous.

The identification of candidate genes from the wild emmer parent that might be responsible for the increased kernel number remains difficult because a large fragment of about 50 million bp (Mbp) was introgressed upstream of Tdurum_contig27976_414 in NIL-U-2B-1 (Table 2, Deblieck et al., 2020). In addition to that, it is not clear which trait(s) are associated with the increased CKN of these plants. Plants with the upper part of the QTL region have an increased MSPL and an increased Sppp (T2 in Table 5). These differences are not significant (Table 5), and therefore, any further conclusions at this point of time remain speculative. However, it might be worth mentioning the presence of the *Ppd-B1* gene from wild emmer on chromosome 2B in NIL-U-2B-1, which has previously been demonstrated to specifically influence the number of seeds per spikelet and not DPH in durum wheat (Arjona et al., 2018). Alternative QTLs in the same region, but close to *Ppd-B1*, have also been identified in wheat in previous studies (Gao et al., 2015; Shi et al., 2017).

Referring to the QTL interval that might be related to MSpL, MSpSp, or MSpSpSp, 111 high-confidence genes were annotated between Gene-1741_103 and Tdurum_contig_68806_677 (Table 2, Table 4, Supplementary Table S9). One of these genes, the ethylene responsive factor (ERF) (TRIDC2BG016990), is a homolog of the well-analyzed Frizzy panicle (FZP) gene (LOC4344233) or Branched Silkless gene (BD1) in rice and maize, respectively (Colombo et al., 1998; Komatsu et al., 2001; Komatsu et al., 2003; Wang et al., 2020; Li et al., 2021). Wheat *fzp* lines were recently shown to have an increased number of spikelets, longer spike length, and reduced TKW (Li et al., 2021). Interestingly, these traits can be attributed to the elite parent allele (Table 4). It might, therefore, be hypothesized that a favorable FZP allele was selected in the course of evolution and introgressed into cultivars such as Uzan. Additional experiments, such as sequence and/or expression analysis, are required to prove or reject this hypothesis.

Finally, a CL-QTL was detected at the lower part of the QTL interval (Table 4). In accordance with this result, Zanke et al. (2014) confirmed that the iSelect marker wsnp_Ex_c6537_11338763 and markers downstream of it are associated with an increased plant height. The authors mention the existence of a gene in wheat that is orthologous to the GIBBERELLIN INSENSITIVE DWARF1 (GID1)-like receptor in rice (Zanke et al., 2014). This region was transferred from G18-16 into the NIL (Deblieck et al., 2020) and might have an impact on the detected CL-QTL. Remarkably, Merchuk-Ovnat et al. (2016) described that an alternative NIL, NIL-U-2B-3, carrying a smaller introgression of G18-16 on chromosome 2B than NIL-U-2B-1, downstream of Ku_c7740_879 (Supplementary Table S1, Merchuck-Ovnat et al., 2016) did not show such a strong increase in culm length relative to Uzan (Supplementary Table S5, Merchuck-Ovnat et al., 2016). Aligning Ku_c7740_879 to the reference genome of Zavitan (Avni et al., 2017) revealed that the marker is located at 164,96 Mbp on chromosome 2BS. We, therefore, hypothesize that the CL-QTL has a size of approximately 26,87 Mbp and ranges from 138.09 (Table 2) to 164,96 Mbp (Merchuck-Ovat et al. .,2016) on chromosome 2BS. This interval contains about 201 annotated high-confidence genes and was transferred into NIL-U-2B-1, but not into NIL-U-2B-3 (Merchuck-Ovnat et al., 2016; Avni et al., 2017). Aligning the rice GID1 gene (LOC4338764) against chromosome 2B of the reference genome of Zavitan (Avni et al., 2017), revealed a homolog candidate gene (TRIDC2BG021600) at position 146.52 Mbp within the interval. However, further fine-mapping of the introgressed G18-16 fragment that flanks wsnp_Ex_c6537_11338763 is required to further narrow down this QTL and to confirm or confute this candidate gene. In addition to this, it should also be taken into consideration that the reduced height gene 4 (Rht4) was described to be located on the long arm of chromosome 2B (Zanke et al., 2014; Wu et al., 2021). Aligning the primer sequences of the most significantly linked SSR marker WMC317 to the reference genome of Zavitan (Avni et al., 2017) revealed that it is located at 762 Mbp and was not transferred from G18-16 into NIL-U-2B-1 (Deblieck et al., 2020). Therefore, the observed differences in plant height cannot be attributed to Rht4.

The main tasks of this study were to identify the smallest sub-QTL region affecting grain yield and to diminish the effects of additional introgressions from the wild emmer parent in NIL-U-2B-1. Both goals were achieved. On the one hand, the delayed DPH and increased CL of NIL-U-2B-1 could be attributed to trans- and linkage drag effects of G18-16 introgressions on chromosome 2A or 2B, respectively. On the other hand, a GY and kernel number increasing G18-16 introgression was identified upstream of Tdurum_contig27976_414. The effect of this on the 50 million Mbp region was confirmed under controlled conditions in all years and under mild drought stress in 2017 (Table 5; Figure 6). Unfortunately, it flanks the QTL interval that was subjected to fine mapping and could not be further narrowed down. However, since the size of the region is much smaller than the original introgression in NIL-U-2B-1 (>400 Mbp) (Deblieck et al., 2020) and since the five selected sub-NILs with this fragment (sub-NILs 1663G, 1688C, 1488A, 1767E, and 1174B) do not show delayed DPH or increased CL, these genotypes might already be crossed with new elite cultivars to establish new NILs. In this respect, the effect of wild emmer introgression might be analyzed in different genomic backgrounds, environments, and years.

In addition to this, it might be useful to further examine the locus in the background of the cv. Uzan. A new fine-mapping approach might, therefore, be accomplished by again backcrossing 1663G, 1688C, 1488A, 1767E, or 1174B with the recurrent parent. Since the region upstream of Tdurum_contig27976_414 is located almost at the end of the chromosome (Table 2; Deblieck et al., 2020), recombinations should appear frequently in this region and fine mapping should be feasible. Finally, repeated trials with different levels of drought stress, Uzan, and the five sub-NILs might reveal to what extent of drought stress the actual yield-increasing effect of this wild emmer fragment persists.

## Data Availability

The datasets presented in this study can be found in online repositories. The data can be found using the following link: https://www.ebi.ac.uk/ena/browser/view/PRJEB53939.

## Abbreviations

CL, culm length; CKN, calculated kernel number; DPH, days from planting to heading; GY, grain yield; HI, harvest index; MSpL, Main Spike Length; MSpTKW, Main Spike Thousand Kernel Weight; Mbp, Million base Pairs; MSpSp, Main Spike Spikelets; MSpSpSp, Main Spike Seeds per Spikelet; NIL, near-isogenic line; PCA, principal component analysis; QTL, quantitative trait loci; SNP, single nucleotide polymorphism; Spp, spikes per plan; TKW, thousand kernel weight; TotDM, total dry matter; WL, water-limited; WW, well-watered.

## References

[B1] Arjona J. M. Royo C. Dreisigacker S. Ammar K. Villegas D. (2018). Effect of ppd-A1 and Ppd-B1 allelic variants on grain number and thousand kernel weight of durum wheat and their impact on final grain yield. Front. Plant Sci. 9, 888. 10.3389/fpls.2018.00888 30008727 PMC6033988

[B2] Avni R. Nave M. Barad O. Baruch K. Twardziok S. O. Gundlach H. (2017). Wild emmer genome architecture and diversity elucidate wheat evolution and domestication. Science 357, 93–97. 10.1126/science.aan0032 28684525

[B3] Below R. Grover-Kopec E. Dilley M. (2007). Documenting drought-related disasters. J. Environ. Dev. 16, 328–344. 10.1177/1070496507306222

[B4] Browning B. L. Browning S. R. (2016). Genotype imputation with millions of reference samples. Am. J. Hum. Genet. 98, 116–126. 10.1016/j.ajhg.2015.11.020 26748515 PMC4716681

[B5] Chen D. Wang S. Cao B. Cao D. Leng G. Li H. (2015). Genotypic variation in growth and physiological response to drought stress and Re-watering reveals the critical role of recovery in drought adaptation in maize seedlings. Front. Plant Sci. 6, 1241. 10.3389/fpls.2015.01241 26793218 PMC4709455

[B6] Colombo L. Marziani G. Masiero S. Wittich P. E. Schmidt R. J. Gorla M. S. (1998). BRANCHED SILKLESSmediates the transition from spikelet to floral meristem duringZea maysear development. Plant J. 16, 355–363. 10.1046/j.1365-313x.1998.00300.x

[B7] Deblieck M. Fatiukha A. Grundman N. Merchuk-Ovnat L. Saranga Y. Krugman T. (2020). GenoTypeMapper: Graphical genotyping on genetic and sequence-based maps. Plant Methods 16, 123. 10.1186/s13007-020-00665-7 32944061 PMC7488165

[B8] Dempewolf H. Baute G. Anderson J. Kilian B. Smith C. Guarino L. (2017). Past and future use of wild relatives in crop breeding. Crop Sci. 57, 1070–1082. 10.2135/cropsci2016.10.0885

[B9] Dhanagond S. Liu G. Zhao Y. Chen D. Grieco M. Reif J. (2019). Non-invasive phenotyping reveals genomic regions involved in pre-anthesis drought tolerance and recovery in spring barley. Front. Plant Sci. 10, 1307. 10.3389/fpls.2019.01307 31708943 PMC6823269

[B45] Elshire R. J. Glaubitz J. C. Sun Q. Poland J. A. Kawamoto K. Buckler E. S. (2011). A robust, simple genotyping-by-sequencing (GBS) approach for high diversity species. PLoS One 6, e19379. 10.1371/journal.pone.0019379 21573248 PMC3087801

[B10] Fahad S. Bajwa A. A. Nazir U. Anjum S. A. Farooq A. Zohaib A. (2017). Crop production under drought and heat stress: Plant responses and management options. Front. Plant Sci. 8, 1147. 10.3389/fpls.2017.01147 28706531 PMC5489704

[B11] Fang Yujie Xiong Lizhong (2015). General mechanisms of drought response and their application in drought resistance improvement in plants. Cell. Mol. Life Sci. 72 (4), 673–689. 10.1007/s00018-014-1767-0 25336153 PMC11113132

[B12] Farooq M. Hussain M. Siddique K. H. M. (2014). Drought stress in wheat during flowering and grain-filling periods. Crit. Rev. Plant Sci. 33, 331–349. 10.1080/07352689.2014.875291

[B47] FAO (2021). World Food and Agriculture-Statistical Yearbook 2021.

[B13] Fatiukha A. Deblieck M. Klymiuk V. Merchuk-Ovnat L. Peleg Z. Ordon F. (2021). Genomic architecture of phenotypic plasticity in response to water stress in tetraploid wheat. Int. J. Mol. Sci. 22, 1723. 10.3390/ijms22041723 33572141 PMC7915520

[B14] Gao F. Wen W. Liu J. Rasheed A. Yin G. Xia X. (2015). Genome-wide linkage mapping of QTL for yield components, plant height and yield-related physiological traits in the Chinese wheat cross zhou 8425B/Chinese spring. Front. Plant Sci. 6, 1099. 10.3389/fpls.2015.01099 26734019 PMC4683206

[B15] Gaspar T. Franck T. Bisbis B. Kevers C. Jouve L. Hausman J. F. (2002). Plant Growth Regul. 37, 263–285. 10.1023/A:1020835304842

[B16] Hackett C. A. (1997). Model diagnostics for fitting QTL models to trait and marker data by interval mapping. Heredity 79, 319–328. 10.1038/hdy.1997.160

[B17] Huang L. Raats D. Sela H. Klymiuk V. Lidzbarsky G. Feng L. (2016). Evolution and adaptation of wild emmer wheat populations to biotic and abiotic stresses. Annu. Rev. Phytopathol. 54, 279–301. 10.1146/annurev-phyto-080614-120254 27296141

[B18] Komatsu M. Chujo A. Nagato Y. Shimamoto K. Kyozuka J. (2003). FRIZZY PANICLE is required to prevent the formation of axillary meristems and to establish floral meristem identity in rice spikelets. Development 130, 3841–3850. 10.1242/dev.00564 12835399

[B19] Komatsu M. Maekawa M. Shimamoto K. Kyozuka J. (2001). The LAX1 and FRIZZY PANICLE 2 genes determine the inflorescence architecture of rice by controlling rachis-branch and spikelet development. Dev. Biol. 231, 364–373. 10.1006/dbio.2000.9988 11237465

[B20] Lane D. R. Coffin D. P. Lauenroth W. K. (2000). Changes in grassland canopy structure across a precipitation gradient. J. Veg. Sci. 11, 359–368. 10.2307/3236628

[B21] Langridge P. Fleury D. (2011). Making the most of 'omics' for crop breeding. Trends Biotechnol. 29, 33–40. 10.1016/j.tibtech.2010.09.006 21030098

[B22] Lesk C. Rowhani P. Ramankutty N. (2016). Influence of extreme weather disasters on global crop production. Nature 529, 84–87. 10.1038/nature16467 26738594

[B23] Li H. (2013). Aligning sequence reads, clone sequences and assembly contigs with BWA-MEM. Available at: https://www.semanticscholar.org/paper/Aligning-sequence-reads%2C-clone-sequences-and-with-Li/74574ee09030e8aadb48fa349eb9b054e2f95ceb .

[B24] Li H. Handsaker B. Wysoker A. Fennell T. Ruan J. Homer N. (2009). The sequence alignment/map format and SAMtools. Bioinformatics 25, 2078–2079. 10.1093/bioinformatics/btp352 19505943 PMC2723002

[B25] Li Y. Li L. Zhao M. Guo L. Guo X. Zhao D. (2021). Wheat FRIZZY PANICLE activates VERNALIZATION1-A and HOMEOBOX4-A to regulate spike development in wheat. Plant Biotechnol. J. 19, 1141–1154. 10.1111/pbi.13535 33368973 PMC8196646

[B26] Merchuk-Ovnat L. Barak V. Fahima T. Ordon F. Lidzbarsky G. A. Krugman T. (2016). Ancestral QTL alleles from wild emmer wheat improve drought resistance and productivity in modern wheat cultivars. Front. Plant Sci. 7, 452. 10.3389/fpls.2016.00452 27148287 PMC4832586

[B27] Merchuk-Ovnat L. Fahima T. Ephrath J. E. Krugman T. Saranga Y. (2017). Ancestral QTL alleles from wild emmer wheat enhance root development under drought in modern wheat. Front. Plant Sci. 8, 703. 10.3389/fpls.2017.00703 28536586 PMC5422550

[B28] Neumann K. Klukas C. Friedel S. Rischbeck P. Chen D. Entzian A. (2015). Dissecting spatiotemporal biomass accumulation in barley under different water regimes using high-throughput image analysis. Plant Cell Environ. 38 (10), 1980–1996. 10.1111/pce.12516 25689277

[B29] Oyewole C. I. (2016). The wheat crop. Anyigab: Kogi State University.

[B30] Pearson K. (1900). X. On the criterion that a given system of deviations from the probable in the case of a correlated system of variables is such that it can be reasonably supposed to have arisen from random sampling. Lond. Edinb. Dublin Philosophical Mag. J. Sci. 50, 157–175. 10.1080/14786440009463897

[B31] Peleg Z. Fahima T. Krugman T. Abbo S. Yakir D. Korol A. B. (2009). Genomic dissection of drought resistance in durum wheat x wild emmer wheat recombinant inbreed line population. Plant Cell Environ. 32, 758–779. 10.1111/j.1365-3040.2009.01956.x 19220786

[B32] Peleg Z. Saranga Y. Suprunova T. Ronin Y. Röder M. S. Kilian A. (2008). High-density genetic map of durum wheat x wild emmer wheat based on SSR and DArT markers. Theor. Appl. Genet. 117, 103–115. 10.1007/s00122-008-0756-9 18437346

[B33] Poole N. Donovan J. Erenstein O. (2021). Viewpoint: Agri-nutrition research: Revisiting the contribution of maize and wheat to human nutrition and health. Food Policy 100 (2021), 101976. 10.1016/j.foodpol.2020.101976 PMC7499093 32963420

[B34] Shapiro S. S. Wilk M. B. (1965). An analysis of variance test for normality (complete samples). Biometrika 52, 591. 10.2307/2333709

[B35] Shi W. Hao C. Zhang Y. Cheng J. Zhang Z. Liu J. (2017). A combined association mapping and linkage analysis of kernel number per spike in common wheat (*Triticum aestivum* L.). Front. Plant Sci. 8, 1412. 10.3389/fpls.2017.01412 28868056 PMC5563363

[B36] Soleimani B. Lehnert H. Keilwagen J. Plieske J. Ordon F. Naseri Rad S. (2020). Comparison between core set selection methods using different Illumina marker platforms: A case study of assessment of diversity in wheat. Front. Plant Sci. 11, 1040. 10.3389/fpls.2020.01040 32754184 PMC7381318

[B37] Takenaka S. Kawahara T. (2012). Evolution and dispersal of emmer wheat (Triticum sp.) from novel haplotypes of Ppd-1 (photoperiod response) genes and their surrounding DNA sequences. Theor. Appl. Genet. 125, 999–1014. 10.1007/s00122-012-1890-y 22639190

[B38] Tukey J. W. (1949). Comparing individual means in the analysis of variance. Biometrics 5, 99–114. 10.2307/3001913 18151955

[B46] United Nations, Department of Economic and Social Affairs, Population Division (2017). World Population Prospects: The 2017 Revision, Key Findings and Advance Tables. Working Paper No. ESA/P/WP/248.

[B39] Wang S.-S. Chung C.-L. Chen K.-Y. Chen R.-K. (2020). A novel variation in the FRIZZLE PANICLE (FZP) gene promoter improves grain number and yield in rice. Genetics 215, 243–252. 10.1534/genetics.119.302862 32152046 PMC7198282

[B40] Wilkinson P. A. Winfield M. O. Barker G. L. A. Tyrrell S. Bian X. Allen A. M. (2016). CerealsDB 3.0: Expansion of resources and data integration. BMC Bioinforma. 17, 256. 10.1186/s12859-016-1139-x PMC4919907 27342803

[B41] Wu Q. Chen Y. Xie J. Dong L. Wang Z. Lu P. (2021). A 36 Mb terminal deletion of chromosome 2BL is responsible for a wheat semi-dwarf mutation. Crop J. 9, 873–881. 10.1016/j.cj.2020.06.015

[B42] Xie W. Nevo E. (2008). Wild emmer: Genetic resources, gene mapping and potential for wheat improvement. Euphytica 164, 603–614. 10.1007/s10681-008-9703-8

[B43] Zanke C. D. Ling J. Plieske J. Kollers S. Ebmeyer E. Korzun V. (2014). Whole genome association mapping of plant height in winter wheat (*Triticum aestivum* L.). PLoS One 9, e113287. 10.1371/journal.pone.0113287 25405621 PMC4236181

[B44] Zhao C. Liu B. Piao S. Wang X. Lobell D. B. Huang Y. (2017). Temperature increase reduces global yields of major crops in four independent estimates. Proc. Natl. Acad. Sci. U. S. A. 114, 9326–9331. 10.1073/pnas.1701762114 28811375 PMC5584412

