# Supplementary material for: Dissection of a grain yield QTL from wild emmer wheat reveals sub-intervals associated with culm length and kernel number
Source: Front Genet. 2022 Oct 19;13:955295. doi: 10.3389/fgene.2022.955295 (PMC9629866; doi:10.3389/fgene.2022.955295)
Supplement: Supplementary file 1 [file DataSheet4.docx]

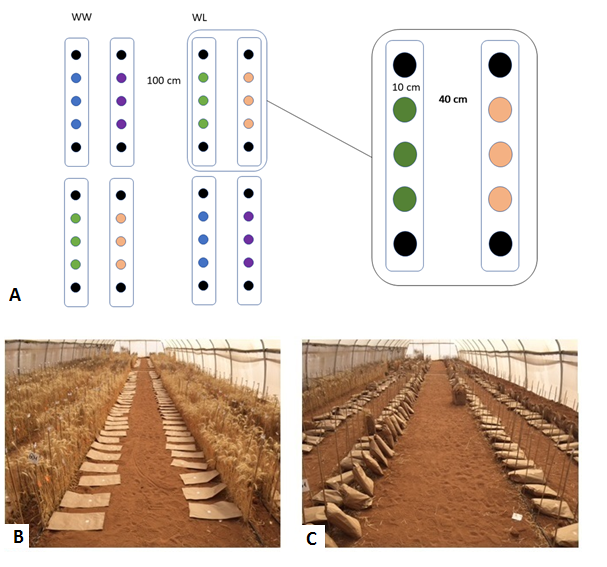
**Figure S2.** Split-block design at the experimental farm of the Hebrew University of Jerusalem in Rehovot, Israel. Top image: (A) A split-plot factorial (genotype x irrigation regime) block design with five replicates was employed; each block consisted of two main plots (for the two irrigation regimes), splited into subplots for genotypes. The rows themselves and the plants were placed 100, 40 and 10 cm apart from each other. Seasonal rainfall was simulated by applying water from planting in December to heading in April/May twice or twice every other week. Bottom photos: Photos of the split block plots before (B) and after harvesting (C) in 2017.
