# Supplementary material for: Dissection of a grain yield QTL from wild emmer wheat reveals sub-intervals associated with culm length and kernel number
Source: Front Genet. 2022 Oct 19;13:955295. doi: 10.3389/fgene.2022.955295 (PMC9629866; doi:10.3389/fgene.2022.955295)
Supplement: Supplementary file 2 [file DataSheet6.docx]

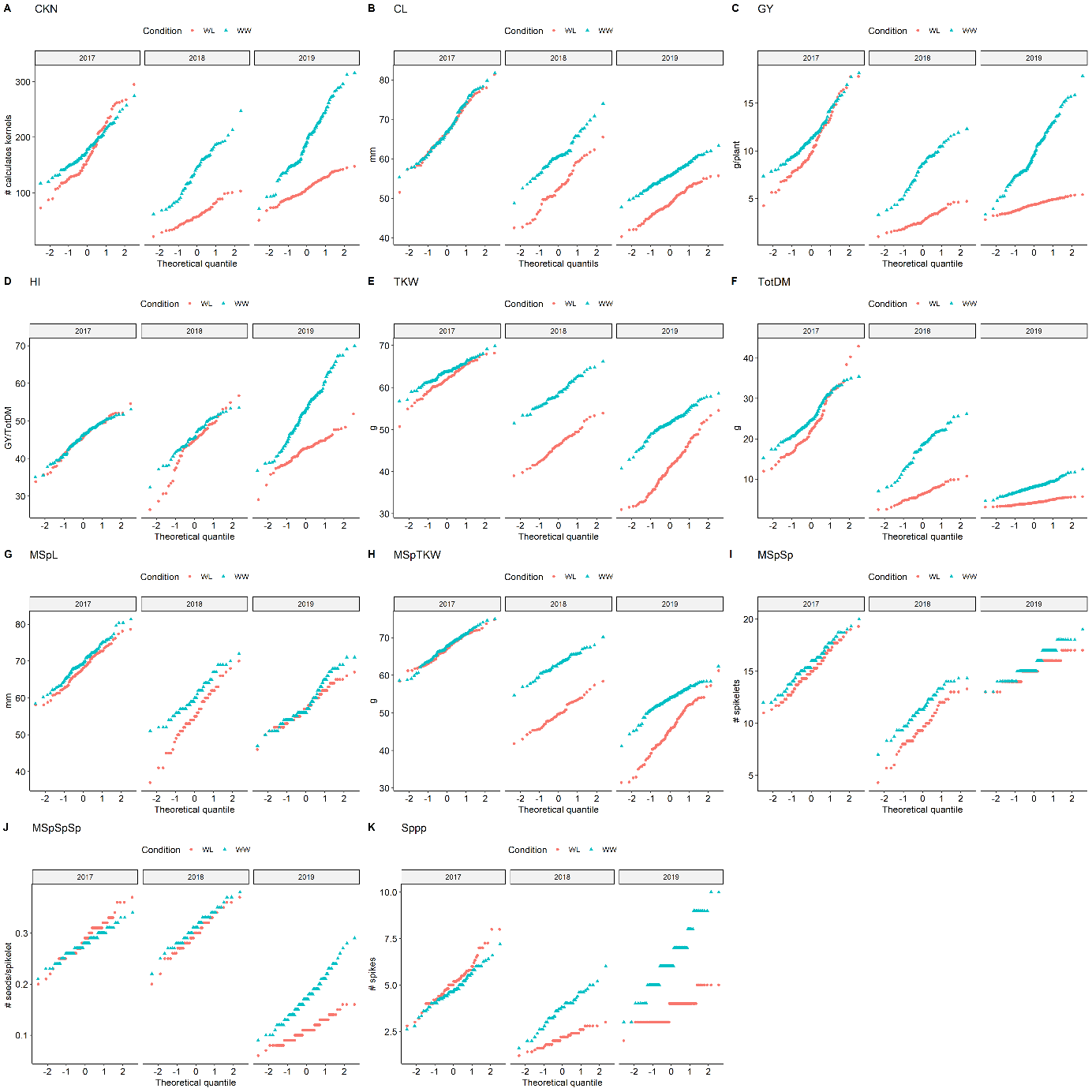


**Figure S4. Quantile-quantile plots of all traits 2017 to 2019.** CKN = Calculated kernel number, CL= Culm Length, GY = Grain yield, HI = Harvest index, TKW = Thousand kernel weight, TotDM= Total Dry Matter, MSpL = Main Spike Length, MSpTKW = Main Spike thousand kernel weight, MSpSp = Main Spike Spikelets, MSpSpSp = Seeds per spikelet (of the main spike), Sppp= Spikelets per plant, WL = Water-limited condition, WW= Well-watered condition
