# Supplementary material for: Dissection of a grain yield QTL from wild emmer wheat reveals sub-intervals associated with culm length and kernel number
Source: Front Genet. 2022 Oct 19;13:955295. doi: 10.3389/fgene.2022.955295 (PMC9629866; doi:10.3389/fgene.2022.955295)
Supplement: Supplementary file 3 [file Table1.DOCX]

**Table S1.** Primer of PCR-based molecular markers

| **iSelect-Marker** | **cM*** | | **Bp*** | | **Forward primer 1*** | **Forward primer 2*** | **Reverse primer** | **Enz.** | **Type** |
| --- | --- | --- | --- | --- | --- | --- | --- | --- | --- |
|  | **Pos.** | **Dis.** | **Pos.** | **Dist.** |  |  |  |  |  |
| Tdurum_contig27976_414 | 32.88 |  | NA |  | CCTTGGTTGCTTGCTCCA | CCTTGGTTGCTTGCTCCG | ATCCGCCGTTTCCTCAGG |  | KASP |
| Kukri_rep_c69803_82 | 35.44 | 2.56 | 77156243 | NA | AGGTTCAGCTGAGAGATGTGA | AGGTTCAGCTGAGAGATGTGG | TCTGAATCTCTCTCCAATAGAAACC |  | KASP |
| BS00010055_51 | 36.48 | 1.04 | 80465477 | 3.309234 | TCAGAGATAGTTCTTTAGCTTCCTT | TCAGAGATAGTTCTTTAGCTTCCTC | GAGGAGTTGAATGATCCTGCT |  | KASP |
| Kukri_c6227780 | 39.36 | 2.88 | 96011570 | 15.546093 | GCGGCCTGTTCAAGTATTACTAA | - | ATTACAAGTTCACCGTCTCCCTA | BSTAPI | CAPS |
| Gene-1741_103 | 40.75 | 1.39 | 97707169 | 1.695599 | GCAGCTTCCACGGTTCATA | GCAGCTTCCACGGTTCATG | AGACGCATACCATGAGCAAA |  | KASP |
| Tdurum_contig30989_79 | 42.15 | 1.4 | 104149860 | 6.442691 | CGCCCTACACCCTTATGTACT | CGCCCTACACCCTTATGTACC | GCCATGGTACCAATCTAGAGTAAC |  | KASP |
| Tdurum_contig_68806_677 | 43.95 | 1.8 | 111425302 | 7.275442 | TGCAACTTTAGTTCAGCTCTTTTT | TGCAACTTTAGTTCAGCTCTTTTC | TCTGTGTTTGTGCTGTATATATGTG |  | KASP |
| Rac875_c2138_474 | 46.81 | 2.86 | 122506008 | 11.080706 | GTGATTGCAGGAGATTGTAA | - | TCCCTTCCCTGCAAACTATGGT | Tsp451 | CAPS |
| Kukri_c46621_14 | 47.86 | 1.05 | 128064567 | 5.558559 | CAGGGTGGACGACGGCAT | CAGGGTGGACGACGGCAC | AAGCACACCAATCCTTTTCAAA |  | KASP |
| wsnp_Ex_c6537_1133876 | 48.55 | 0.69 | 138099492 | 10.034925 | ACTATCCAGCACCGGAGAAT | ACTATCCAGCACCGGAGAAC | GTGCACCCAATTCAGCGTT |  | KASP |

***)** Centimorgan (cM), Basepair (BP), Position (Pos), Distance (Dist). No physical position was obtained for the marker Tdurum_contig27976_414. Genetic positions were obtained from Fatiukha et al. (2021).
