# Supplementary material for: Dissection of a grain yield QTL from wild emmer wheat reveals sub-intervals associated with culm length and kernel number
Source: Front Genet. 2022 Oct 19;13:955295. doi: 10.3389/fgene.2022.955295 (PMC9629866; doi:10.3389/fgene.2022.955295)
Supplement: Supplementary file 4 [file DataSheet3.docx]

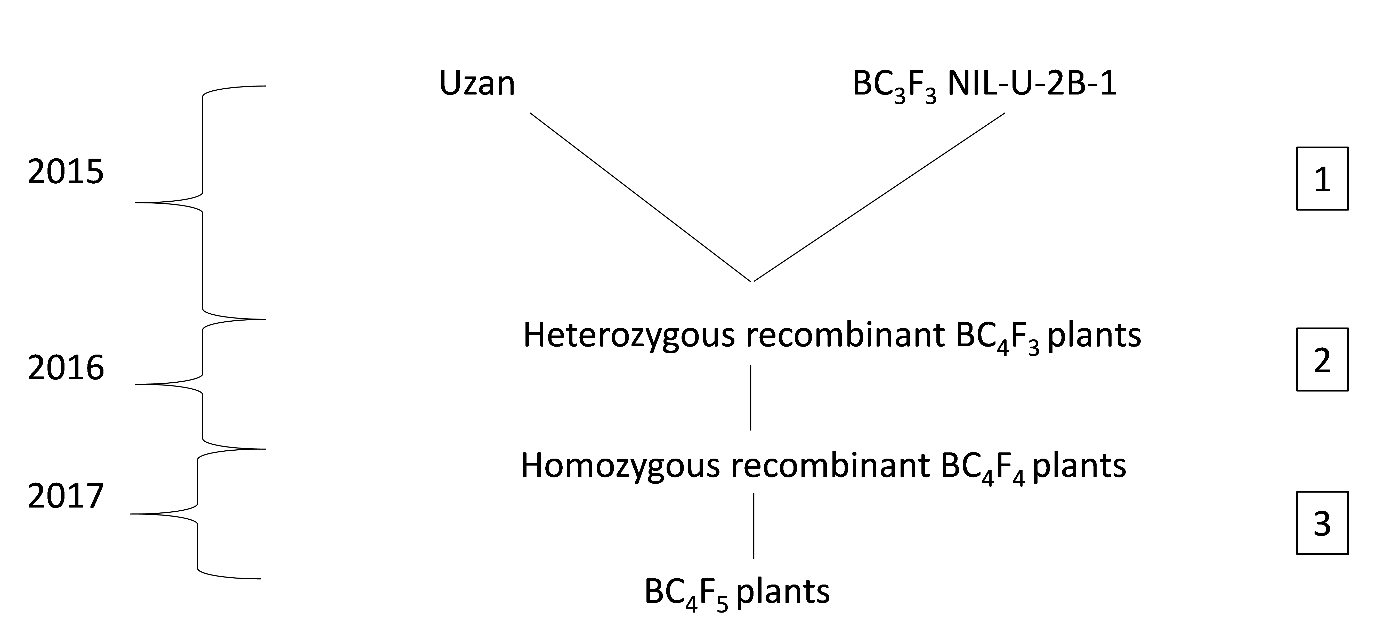


**Figure S1. Establishment of sub-NILs** 1) BC_4_F_3_ plants were established by crossing the BC_3_F_3_ near isogenic line NIL-U-2B-1 (pollinator) with the elite durum parent Uzan. 2) Subsequently, BC_4_F_3_ plants were genotyped to identify heterozygous recombinant plants with recombination events scattered along the target interval and BC_4_F_4_ single seed descendants of BC_4_F_3_ plants were screened to identify homozygous recombinant plants. 3) Finally homozygous recombinant BC_4_F_4_ plants were used to produce BC_4_F_5_ seeds for the phenotypic experiments in 2017, 2018 and 2019.
