# Supplementary figures and images for: Dissection of a grain yield QTL from wild emmer wheat reveals sub-intervals associated with culm length and kernel number
Source: Front Genet. 2022 Oct 19;13:955295. doi: 10.3389/fgene.2022.955295 (PMC9629866; doi:10.3389/fgene.2022.955295)

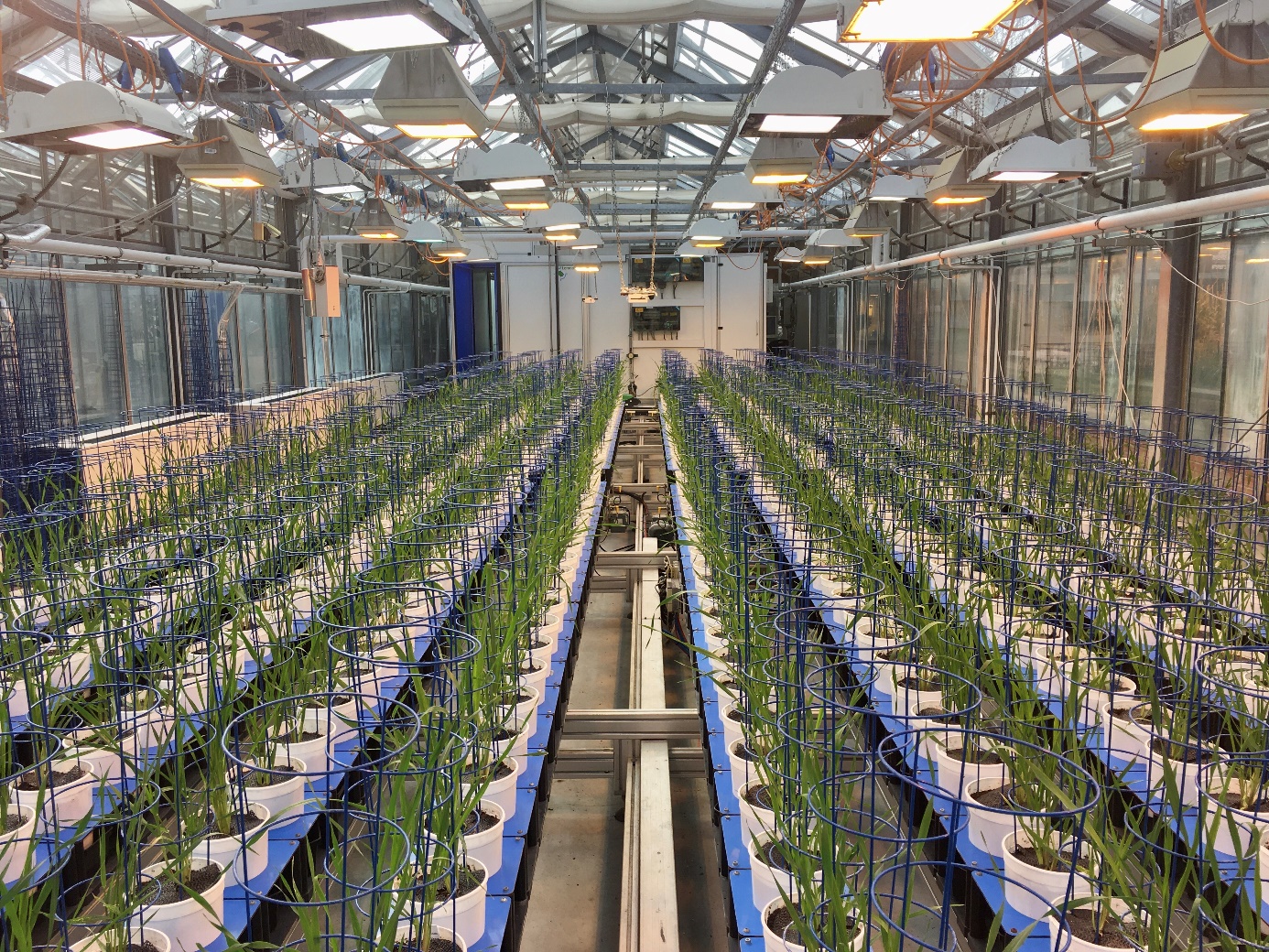


**Figure S3. Picture of the Lemnatec fascility at the IPK in Gatersleben, Germany**

Supplement: Supplementary file 5 [file DataSheet5.docx]
