# Supplementary material for: Dissection of a grain yield QTL from wild emmer wheat reveals sub-intervals associated with culm length and kernel number
Source: Front Genet. 2022 Oct 19;13:955295. doi: 10.3389/fgene.2022.955295 (PMC9629866; doi:10.3389/fgene.2022.955295)
Supplement: Supplementary file 6 [file Table2.DOCX]

**Table S2.** Standard Real Time program for KASP-markers

|  | | Start |  | 15 cycles |  | 40 cycles |  | End |
| --- | --- | --- | --- | --- | --- | --- | --- | --- |
| Step | | Step1 | Step2 | Step1 | Step2 | Step1 | Step2 | Step1 |
| Time | | 1 Min | 10 Min | 20 s | 1 Min | 20 S | 1 min | 1 min |
| Marker | Tdurum_contig27976_414 | 30 °C | 94 °C | 94 °C | 61,0 °C | 94 °C | 57.2 °C | 30 °C |
|  | Kukri_c46621_143 | 30 °C | 94 °C | 94 °C | 61,0 °C | 94 °C | 57.2 °C | 30 °C |
|  | BS00010055_51 | 30 °C | 94 °C | 94 °C | 61,0 °C | 94 °C | 57 °C | 30 °C |
|  | RAC875_c3302_1411 | 30 °C | 94 °C | 94 °C | 61,0 °C | 94 °C | 57.2 °C | 30 °C |
|  | Tdurum_contig30989_79 | 30 °C | 94 °C | 94 °C | 61,0 °C | 94 °C | 57 °C | 30 °C |
|  | Kukri_c46621_143 | 30 °C | 94 °C | 94 °C | 61,0 °C | 94 °C | 57 °C | 30 °C |
|  | wsnp_Ex_c6537_11338763 | 30 °C | 94 °C | 94 °C | 61,0 °C | 94 °C | 57.3 °C | 30 °C |
