# Supplementary material for: Dissection of a grain yield QTL from wild emmer wheat reveals sub-intervals associated with culm length and kernel number
Source: Front Genet. 2022 Oct 19;13:955295. doi: 10.3389/fgene.2022.955295 (PMC9629866; doi:10.3389/fgene.2022.955295)
Supplement: Supplementary file 7 [file Table5.docx]

| **Condition** |  | **X̅**** | | | **S**** | | | **Shapiro Wilk test (p-value)** | | | **ANOVA**  **P-value** |
| --- | --- | --- | --- | --- | --- | --- | --- | --- | --- | --- | --- |
|  | **Trait*** | **2017** | **2018** | **2019** | **2017** | **2018** | **2019** | **2017** | **2018** | **2019** |  |
| **Water-limited treatment** | CL | 66.88 | 52.80 | 48.67 | 4.48 | 2.75 | 2.77 | 0.48 | 0.08 | 0.59 | 0.00 |
|  | MSpL*** | 4.22 | 4.01 | 4.05 | 0.05 | 0.06 | 0.06 | 0.67 | 0.69 | 0.14 | 0.00 |
|  | MSpsp | 15.05 | 9.57 | 15.21 | 1.48 | 1.01 | 0.73 | 0.18 | 0.12 | 0.40 | 0.86 |
|  | MSpSpSp | 0.29 | 0.30 | 0.11 | 0.03 | 0.03 | 0.01 | 0.66 | 0.66 | 0.55 | 0.00 |
|  | MSpTKW | 66.92 | 49.91 | 44.95 | 2.28 | 2.38 | 5.06 | 1.00 | 0.63 | 0.36 | 0.00 |
|  | TotDM | 22.98 | 6.34 | 4.21 | 2.53 | 1.01 | 0.48 | 0.38 | 0.74 | 0.54 | 0.00 |
|  | GY | 10.39 | 2.75 | 4.33 | 1.25 | 0.44 | 0.30 | 0.65 | 0.30 | 0.13 | 0.00 |
|  | HI | 45.51 | 43.70 | NA | 2.93 | 3.04 | NA | 0.46 | 0.12 | 0.07 | 0.00 |
|  | Sppp*** | 1.63 | 0.75 | 1.29 | 0.07 | 0.10 | 0.14 | 0.09 | 0.00 | 0.09 | 0.01 |
|  | GYpSp*** | 0.68 | 0.25 | 0.18 | 0.08 | 0.09 | 0.10 | 0.47 | 0.55 | 0.52 | 0.00 |
|  | TKW | 61.78 | 46.38 | 40.64 | 2.75 | 2.83 | 4.58 | 0.54 | 0.56 | 0.67 | 0.00 |
|  | CKN*** | 5.12 | 4.07 | 4.68 | 0.12 | 0.19 | 0.14 | 0.80 | 0.17 | 0.28 | 0.00 |
| **Well-watered treatment** | CL | 67.63 | 61.08 | 55.79 | 5.20 | 3.19 | 2.09 | 0.72 | 0.50 | 0.07 | 0.04 |
|  | MSpL*** | 4.25 | 4.10 | 4.07 | 0.04 | 0.05 | 0.08 | 0.65 | 0.87 | 0.07 | 0.00 |
|  | MSpsp | 15.70 | 11.43 | 15.76 | 1.33 | 1.02 | 0.99 | 0.09 | 0.41 | 0.24 | 0.94 |
|  | MSpSpSp | 0.28 | 0.31 | 0.17 | 0.02 | 0.03 | 0.02 | 0.30 | 0.78 | 0.99 | 0.00 |
|  | MSpTKW | 67.38 | 63.01 | 53.38 | 2.39 | 1.93 | 2.04 | 0.53 | 0.61 | 0.28 | 0.00 |
|  | TotDM | 25.43 | 17.44 | 8.18 | 2.85 | 2.62 | 1.15 | 1.00 | 0.29 | 0.09 | 0.00 |
|  | GY | 11.59 | 8.10 | 10.08 | 1.38 | 1.08 | 2.39 | 0.47 | 0.81 | 0.10 | 0.04 |
|  | HI | 45.59 | 45.75 | 53.27 | 3.53 | 2.92 | 6.83 | 0.39 | 0.55 | 0.32 | 0.00 |
|  | Sppp*** | 1.56 | 1.31 | 1.86 | 0.10 | 0.12 | 0.15 | 0.53 | 0.06 | 0.28 | 0.00 |
|  | GYpSp*** | 0.87 | 0.80 | 0.44 | 0.08 | 0.07 | 0.12 | 0.54 | 0.23 | 0.10 | 0.00 |
|  | TKW | 63.57 | 58.94 | 51.49 | 1.57 | 2.72 | 1.77 | 0.95 | 0.75 | 0.42 | 0.00 |
|  | CKN*** | 5.18 | 4.93 | 5.25 | 0.11 | 0.16 | 0.23 | 0.60 | 0.43 | 0.03 | 0.34 |

**Table S6.** Analysis of variance (ANOVA) of phenotypic traits under controlled and drought stress conditions.

* CL, culm length; CKN, calculated kernel number; GY, grain yield; GypSp, grain yield per spike; HI, harvest index; TKW, Thousand kernel weight; MSPTKW, Main Spike Thousand Kernel Weight; MSpL, Main Spike Length; MSPSP, Main Spike Spikelets; MSpSpSp, Main Spike Seeds per Spikelet; Sppp, Spikes per plant, ** X̅, mean value; s, standard deviation *** These values were logtransformed due to the reason that the initial datasets were not normally distributed.
