# Supplementary material for: Dissection of a grain yield QTL from wild emmer wheat reveals sub-intervals associated with culm length and kernel number
Source: Front Genet. 2022 Oct 19;13:955295. doi: 10.3389/fgene.2022.955295 (PMC9629866; doi:10.3389/fgene.2022.955295)
Supplement: Supplementary file 8 [file Table3.DOCX]

**Table S3.** PCR-programs for the CAPS markers Kukri_c6227780 and Rac875_c2138_474

| **PCR Step** | **Temperature °C** | **Duration** |
| --- | --- | --- |
| Denatururation | 94 °C | 1 min |
| Annealing | 58 °C | 45 s |
| Extension | 72 °C | 45s |

*The CAPS markers Kukri_c6227780 and Rac875_c2138_474 will lead to products of about 448 and 342 basepairs respectively
