# Supplementary material for: Dissection of a grain yield QTL from wild emmer wheat reveals sub-intervals associated with culm length and kernel number
Source: Front Genet. 2022 Oct 19;13:955295. doi: 10.3389/fgene.2022.955295 (PMC9629866; doi:10.3389/fgene.2022.955295)
Supplement: Supplementary file 9 [file Table4.docx]

**Table S5.** **Paired t-tests for phenotypic traits under controlled and drought stress conditions**

| Year | Condition | Well-watered treatment | | | Water-limited treatment | | | Ttest(pv-) |
| --- | --- | --- | --- | --- | --- | --- | --- | --- |
|  | Traits* | **X̅** | **s** | S.W. pv | **X̅** | **s** | S.W. pv |  |
| 2017 | ~~CL~~ | ~~67.63~~ | ~~5.20~~ | ~~0.48~~ | ~~66.88~~ | ~~4.48~~ | ~~0.72~~ | ~~0.34~~ |
|  | MSpL*** | 4.25 | 0.04 | 0.67 | 4.22 | 0.05 | 0.65 | 0.00 |
|  | MSpSp | 15.70 | 1.33 | 0.18 | 15.05 | 1.48 | 0.09 | 0.00 |
|  | MSpSpSp | 0.28 | 1.33 | 0.66 | 0.29 | 1.48 | 0.30 | 0.02 |
|  | ~~MSpTKW~~ | ~~67.38~~ | ~~2.39~~ | ~~1.00~~ | ~~66.92~~ | ~~2.28~~ | ~~0.53~~ | ~~0.29~~ |
|  | GY | 11.59 | 1.38 | 0.65 | 10.39 | 1.25 | 0.47 | 0.00 |
|  | ~~HI~~ | ~~45.59~~ | ~~3.53~~ | ~~0.46~~ | ~~45.51~~ | ~~2.93~~ | ~~0.39~~ | ~~0.88~~ |
|  | Sppp*** | 1.56 | 0.10 | 0.09 | 1.63 | 0.07 | 0.53 | 0.01 |
|  | GYpSp*** | 0.87 | 0.08 | 0.47 | 0.68 | 0.08 | 0.54 | 0.00 |
|  | TKW | 63.57 | 1.57 | 0.54 | 61.78 | 2.75 | 0.95 | 0.01 |
|  | CKN*** | 5.18 | 0.11 | 0.80 | 5.12 | 0.12 | 0.60 | 0.05 |
| 2018 | CL | 61.08 | 3.19 | 0.08 | 52.80 | 2.75 | 0.50 | 0.00 |
|  | MSpL*** | 4.10 | 0.05 | 0.69 | 4.01 | 0.06 | 0.87 | 0.00 |
|  | MSpSp | 11.43 | 1.02 | 0.12 | 9.57 | 1.01 | 0.41 | 0.00 |
|  | MSpSpSp | 0.31 | 1.02 | 0.66 | 0.30 | 1.01 | 0.78 | 0.01 |
|  | MSpTKW | 63.01 | 1.93 | 0.63 | 49.91 | 2.38 | 0.61 | 0.00 |
|  | GY | 8.10 | 1.08 | 0.30 | 2.75 | 0.44 | 0.81 | 0.00 |
|  | HI | 45.75 | 2.92 | 0.12 | 43.70 | 3.04 | 0.55 | 0.06 |
|  | *Sppp**** | *1.31* | *0.12* | *0.00* | *0.75* | *0.10* | *0.06* | *0.00* |
|  | GYpSp*** | 0.80 | 0.07 | 0.55 | 0.25 | 0.09 | 0.23 | 0.00 |
|  | TKW | 58.94 | 2.72 | 0.56 | 46.38 | 2.83 | 0.75 | 0.00 |
|  | CKN*** | 4.93 | 0.16 | 0.17 | 4.07 | 0.19 | 0.43 | 0.00 |
| 2019 | CL | 55.79 | 2.09 | 0.59 | 48.67 | 2.77 | 0.07 | 0.00 |
|  | MSpL*** | 4.07 | 0.08 | 0.14 | 4.05 | 0.06 | 0.07 | 0.12 |
|  | MSpSp | 15.76 | 0.99 | 0.40 | 15.21 | 0.73 | 0.24 | 0.00 |
|  | MSpSpSp | 0.17 | 0.99 | 0.55 | 0.11 | 0.73 | 0.99 | 0.00 |
|  | MSpTKW | 53.38 | 2.04 | 0.36 | 44.95 | 5.06 | 0.28 | 0.00 |
|  | GY | 10.08 | 2.39 | 0.13 | 4.33 | 0.30 | 0.10 | 0.00 |
|  | HI | 53.27 | 6.83 | 0.07 | NA |  | 0.32 | 0.00 |
|  | Sppp*** | 1.86 | 0.15 | 0.09 | 1.29 | 0.14 | 0.28 | 0.00 |
|  | GYpSp*** | 0.44 | 0.12 | 0.52 | 0.18 | 0.10 | 0.10 | 0.00 |
|  | TKW | 51.49 | 1.77 | 0.67 | 40.64 | 4.58 | 0.42 | 0.00 |
|  | CKN*** | 5.25 | 0.23 | 0.28 | 4.68 | 0.14 | 0.03 | 0.00 |

* CL, culm length; CKN, calculated kernel number; GY, grain yield; GypSp, grain yield per spike; HI, harvest index; TKW, Thousand kernel weight; MSPTKW, Main Spike Thousand Kernel Weight; MSpL, Main Spike Length; MSPSP, Main Spike Spikelets; MSpSpSp, Main Spike Seeds per Spikelet; Sppp, Spikes per plant; p-values that were significant (p<0.05 were highlighted in bold); s, standard deviation ** paired t-tests – significant p-values <0.05 are highlighted in bold. *** These values were logtransformed due tot he reason that the initial datasets were not normally distributed. Please note that CKN and Sppp in the years 2018 and 2019 remained not normally distributed after log transormation and were therefore written in italics. No significant drought stress effectes were detected in 2017 for CL, MSpTKW and HI. Therefore this data was not used for QTL-detection.
