# Supplementary material for: Dissection of a grain yield QTL from wild emmer wheat reveals sub-intervals associated with culm length and kernel number
Source: Front Genet. 2022 Oct 19;13:955295. doi: 10.3389/fgene.2022.955295 (PMC9629866; doi:10.3389/fgene.2022.955295)
Supplement: Supplementary file 10 [file Table6.docx]

| Screenhouse | Year | 2017 | | | | | | 2018 | | | | |
| --- | --- | --- | --- | --- | --- | --- | --- | --- | --- | --- | --- | --- |
|  | Number of days in the months* | 9 | 23 | 28 | 31 | 30 | 17 | 31 | 28 | 31 | 30 | - |
|  | Month | Days of the month | January | February | March | April | May | January | February | March | April | May |
| Unit: Hobo 9. | Minimum average | 1 - 10 |  | 7.60 | 10.96 | 13.34 | 16.49 | 7.18 | 6.78 | 9.67 | 11.92 | - |
|  |  | 11 - 20 | 8.55 | 8.90 | 12.39 | 14.40 | 18.23 | 8.48 | 9.08 | 8.78 | 13.65 | - |
|  |  | 21 - 30 | 8.68 | 7.47 | 11.90 | 14.81 |  | 7.38 | 8.78 | 9.97 | 12.88 | - |
|  | Maximum average | 1 - 10 |  | 30.86 | 35.04 | 39.00 | 46.29 | 36.95 | 40.30 | 44.21 | 42.16 | - |
|  |  | 11 - 20 | 29.64 | 31.25 | 34.08 | 39.89 | 49.46 | 37.60 | 40.65 | 49.77 | 46.85 | - |
|  |  | 21 - 30 | 30.41 | 32.38 | 35.59 | 42.85 |  | 41.34 | 43.12 | 51.72 | 64.91 | - |
|  | Average | 1 - 10 |  | 16.17 | 19.61 | 23.38 | 28.66 | 17.30 | 18.53 | 22.13 | 23.57 | - |
|  |  | 11 - 20 | 15.85 | 15.88 | 19.92 | 24.39 | 30.90 | 17.99 | 18.81 | 21.78 | 26.32 | - |
|  |  | 21 - 30 | 15.97 | 17.03 | 20.53 | 26.69 |  | 16.17 | 20.09 | 25.60 | 28.23 | - |
| . Unit: Hobo 8. | Minimum average | 1 - 10 |  | 7.02 | 7.38 | 11.63 | 14.52 | 7.38 | 6.78 | 9.57 | 12.01 | - |
|  |  | 11 - 20 | 7.87 | 7.03 | 8.41 | 11.63 | 14.69 | 8.48 | 8.98 | 8.88 | 13.56 | - |
|  |  | 21 - 30 | 7.78 | 7.26 | 11.16 | 13.90 |  | 7.38 | 8.68 | 10.06 | 13.08 | - |
|  | Maximum average | 1 - 10 |  | 13.98 | 19.47 | 17.41 | 22.91 | 37.38 | 40.88 | 46.59 | 45.20 | - |
|  |  | 11 - 20 | 11.60 | 16.11 | 19.47 | 21.16 | 22.94 | 39.05 | 42.28 | 52.87 | 49.09 | - |
|  |  | 21 - 30 | 14.23 | 14.34 | 17.53 | 22.80 |  | 41.69 | 45.20 | 53.17 | 67.11 | - |
|  | Average | 1 - 10 |  | 9.94 | 11.05 | 13.98 | 17.81 | 17.58 | 18.84 | 22.74 | 24.07 | - |
|  |  | 11 - 20 | 9.59 | 10.71 | 12.21 | 15.22 | 18.66 | 18.34 | 19.14 | 22.36 | 26.74 | - |
|  |  | 21 - 30 | 10.63 | 9.92 | 13.35 | 16.80 |  | 16.49 | 20.56 | 26.14 | 28.86 | - |

**Table S8. Average temperatures in 2017 and 2018 at the experimental farm of the Hebrew University of Jerusalem in Rehovot.**

* The maximum, minimum and average temperatures were measured on a daily base.
